# Design and selection of optimal ErbB-targeting bispecific antibodies in pancreatic cancer

**DOI:** 10.3389/fimmu.2023.1168444

**Published:** 2023-04-20

**Authors:** Emilia Rabia, Véronique Garambois, Christine Dhommée, Christel Larbouret, Laurie Lajoie, Yoan Buscail, Gabriel Jimenez-Dominguez, Sylvie Choblet-Thery, Emmanuelle Liaudet-Coopman, Martine Cerutti, Marta Jarlier, Patrice Ravel, Laurent Gros, Nelly Pirot, Gilles Thibault, Eugene A. Zhukovsky, Pierre-Emmanuel Gérard, André Pèlegrin, Jacques Colinge, Thierry Chardès

**Affiliations:** ^1^ IRCM, Institut de Recherche en Cancérologie de Montpellier, INSERM, Université de Montpellier, Institut régional du Cancer de Montpellier, Montpellier, France; ^2^ GICC, Groupe Innovation et Ciblage Cellulaire, Université de Tours, Tours, France; ^3^ Réseau d’Histologie Expérimentale de Montpellier, BioCampus, Université de Montpellier, UAR3426 CNRS-US09 INSERM, Montpellier, France; ^4^ Plateforme Bacfly, Baculovirus et Thérapie, BioCampus, UAR3426 CNRS-US09 INSERM, Saint-Christol-Lèz Alès, France; ^5^ ICM, Institut régional du Cancer de Montpellier, Montpellier, France; ^6^ CNRS, Centre National de la Recherche Scientifique, Paris, France; ^7^ Biomunex Pharmaceuticals, Incubateur Paris Biotech santé, Hopital Cochin, Paris, France

**Keywords:** ErbB, systems biology, antibody, bispecific, pancreatic cancer, ADCC, signaling, phosphoproteome

## Abstract

The ErbB family of receptor tyrosine kinases is a primary target for small molecules and antibodies for pancreatic cancer treatment. Nonetheless, the current treatments for this tumor are not optimal due to lack of efficacy, resistance, or toxicity. Here, using the novel BiXAb™ tetravalent format platform, we generated bispecific antibodies against EGFR, HER2, or HER3 by considering rational epitope combinations. We then screened these bispecific antibodies and compared them with the parental single antibodies and antibody pair combinations. The screen readouts included measuring binding to the cognate receptors (mono and bispecificity), intracellular phosphorylation signaling, cell proliferation, apoptosis and receptor expression, and also immune system engagement assays (antibody-dependent cell-mediated cytotoxicity and complement-dependent cytotoxicity). Among the 30 BiXAbs™ tested, we selected 3Patri-1Cetu-Fc, 3Patri-1Matu-Fc and 3Patri-2Trastu-Fc as lead candidates. The in vivo testing of these three highly efficient bispecific antibodies against EGFR and HER2 or HER3 in pre-clinical mouse models of pancreatic cancer showed deep antibody penetration in these dense tumors and robust tumor growth reduction. Application of such semi-rational/semi-empirical approach, which includes various immunological assays to compare pre-selected antibodies and their combinations with bispecific antibodies, represents the first attempt to identify potent bispecific antibodies against ErbB family members in pancreatic cancer.

## Introduction

Members of the ErbB family (EGFR, HER2, HER3, and HER4) of receptor tyrosine kinases (RTK) are key targets in cancer therapy. RTKs act by linking the tumor microenvironment to intracellular molecular networks that influence cancer cell behavior. Multiple ligands, such as Epidermal Growth Factor (EGF; EGFR ligand), and neuregulin 1 (NRG1; HER3 ligand), initiate signaling by binding to receptors and inducing their dimerization (1). The dimer composition influences the nature of intracellular signaling and cellular responses as well as receptor trafficking and degradation. Many RTKs are activated by various ligands, and induce multiple downstream pathways with feedback loops and cross-talks, such as the ERK and AKT pathways (2). Abnormal HER signaling can be caused by ligand or receptor overexpression, or mutation-related constitutive activity that may lead to drug resistance (3, 4).

The complexity of RTK activation and of the downstream pathway cross-talk and the intrinsic signal transduction robustness (5–9) make it difficult to reduce RTK activity by inhibiting a single RTK or a unique downstream kinase. We (10–12) and others (13, 14) proposed to combine monoclonal antibodies (mAbs) against different RTKs or different epitopes of the same receptor to overcome acquired resistance or to circumvent signaling robustness. We found that the combination of cetuximab (anti-EGFR mAb) and trastuzumab (anti-HER2 mAb) is efficient against pancreatic cancer (10–12), a disease with unmet medical needs and poor prognosis (15). This preclinical work, confirmed by other research groups (13, 14, 16), led to the initiation of the THERAPY phase I/II clinical trial in patients with metastatic pancreatic cancer who progressed on gemcitabine. This trial showed that the cetuximab-trastuzumab combination stabilizes the disease in 27% of patients, without objective response but with a positive correlation between skin toxicity and progression-free survival (17). Other studies also showed that homo-combinations of two antibodies against EGFR (18–21), HER2 (22–24), or HER3 (25), and hetero-combinations of antibodies against EGFR and HER2 (10–14, 16, 26), or EGFR and HER3 (27, 28) induce anti-tumor activity through accelerated degradation of the targeted receptors, enhanced antibody-dependent cell-mediated cytotoxicity (ADCC) and reduction of dimer formation, thus bypassing the resistance to treatment induced by monotherapy. They also maintain the anti-tumor activity despite the presence of mutations in EGFR extracellular domain (ECD) that might impair antibody binding (29). Similarly, mixtures of three antibodies against HER2 (30) or EGFR (Sym004) (31) resulted in therapeutic benefits in HER2-amplified breast cancer and lung cancer, respectively. In experimental models, oligoclonal cocktails of three (32, 33) to six (34, 35) antibodies against EGFR, HER2 and HER3 increased the anti-tumor effect with blockade of the intracellular ERK and AKT signaling pathways and accelerated receptor degradation. These pre-clinical results led to more than 20 phase II/III clinical trials in patients with cancer to test ErbB antibody associations (36). However, due to RTK signaling complexity, it is very difficult to predict the effects (synergy or additivity) of antibody combinations against a specific tumor.

In recent years, bispecific antibodies (BsAbs) (37) have been developed to bridge immune cells to tumor cells to increase cancer cell killing and to dually target RTKs. Some examples of BsAbs against ErbB members are anbenitamab (KN026) (38) and zanidatamab (ZW25) (39) (against HER2/HER2), duligotuzumab (MEHD7945A) (40) and Izalontamab (SI-B001) (41) (against EGFR/HER3), and MM-111 (42) and zenocutuzumab (MCLA-128) (43) (against HER2/HER3). These BsAbs have been evaluated in clinical trials, but with inconclusive results in many cases. Currently, MCLA-128 is tested in clinical trials in patients with HER2-amplified and HER2-low/estrogen receptor-positive breast cancer, and in phase 1/2 trials in patients with NRG1-positive pancreatic and lung cancer (44). The anti-HER2/HER2 BsAbs KN026 and ZN25 are evaluated in a phase 1 trial (HER2-amplified breast and gastric cancer) and in a phase 2 trial (biliary tract cancer) (45–47). The anti-EGFR/HER3 BsAb SI-B001 is included in phase 2/3 trials in lung cancer (41). Moreover, anti-EGFR/HER2 (48, 49), anti-HER2/HER3 (50, 51) and anti-EGFR/HER3 (52, 53) BsAbs, and a tetravalent anti-EGFR/HER2/HER3/VEGF antibody (54) have been evaluated in pre-clinical studies.

Some authors used mathematical modeling of antibody combinations, particularly by taking into account feedback loops in RTK signaling that can intrinsically cause resistance to single therapies (5, 6). This led to the identification of the BsAbs MM-111 (against HER2/HER3) (42) and istiratumab (MM-141) (against IGF-1R/HER3) (55). These models took into account signaling networks and sometimes internalization processes, but they did not integrate them with antibody-mediated immune mechanisms, such as ADCC or complement-dependent cytotoxicity (CDC). However, it has been shown that clinically-approved antibodies against EGFR or HER2 act predominantly through these immune mechanisms to reduce tumor growth (56, 57). Yet, the existing models essentially ignore the influence of ADCC/CDC on receptor dynamics when combined therapies include antibodies. Moreover, no global analysis evaluated whether the cell signaling effects, RTK degradation, and immune-induced effects induced by BsAbs targeting ErbB family members are different from those induced by single mAbs or mAb combinations. BsAbs could locally show increased avidity for two receptors and a natural tendency to favor clustering of receptor lattices, thus improving natural killer (NK) cell recognition and ADCC.

In this study, we performed a rational exploration of potential BsAb designs to target pancreatic cancer. To provide a global picture, we considered six therapeutic mAbs against EGFR, HER2 and HER3 (two *per* receptor) that target different epitopes and competing or not with the ligand. We tested these antibodies as monotherapy (1MAbs), in combination (2MAbs), and as BsAbs. We produced the BsAbs using the IgG-based tetravalent BiXAb™ platform. We included two binding sites (two tandem Fab domains on each arm) for each receptor, thus maintaining bivalence, and a functional Fc domain. We designed 30 BiXAb™ by considering both external (N-terminal) and internal positions for each Fab domain on the BiXAb™ arms, and screened them *in vitro* in pancreatic cancer and patient-derived xenograft (PDX)-derived cell lines benchmarking them to the corresponding 1MAbs and 2MAbs. Through successive selections of the most promising molecules based on their effects on downstream kinase signaling, cell proliferation and apoptosis, receptor degradation, and immune system engagement, together with network modeling, we identified three lead BiXAb™ and compared them to 1MAbs and 2MAbs in pre-clinical models of pancreatic cancer.

## Methods

### Cell lines and culture

The BxPC3, Sw1990, AsPC-1, and CFPAC pancreatic cancer cell lines and the SKBR3 breast cancer cell line were from ATCC (Rockville, MD) and were cultured following the ATCC recommendations. The two PDXs (P4604 and P2846) were generated from resected hepatic and peritoneum metastatic samples, respectively, from two patients with pancreatic cancer treated with gemcitabine (P4604) or untreated at surgery time (P2846) (PDX Platform, Institut de Recherche en Cancérologie de Montpellier) (58). The PDX cell line C-PDX P4604 was derived from the PDX P4604. The gemcitabine-resistant cell line BxPC3-GR was generated by exposure to progressively increasing doses of gemcitabine, as previously described (58). All cell lines and the PDXs express EGFR at high level (between 150,000 and 350,000 receptors/cell), HER2 at intermediate level (between 6,000 and 78,000 receptors/cell), and HER3 at low level (between 5,000 and 20,000 receptors/cell) (26, 58, 59), except the SKBR3 cell line that is HER2^high^ (>1 million receptor/cell) (59). The CD16/FcγRIII (158V allotype)-transfected human NK92 cell line was cultured in complete RPMI medium supplemented with 100 IU/ml interleukin 2, as previously described (60).

### Recombinant proteins and antibodies

Tag-free recombinant human HER2 (SB #10004-HCCH) and HER3 (SB #10201-HCCH) ECD, and His-tagged recombinant human EGFR (SB #10001-H08H), HER2 (SB #10004-H08H) and HER3 (SB #10201-H08H) ECD were purchased from Sino Biological Inc (China). Tag-free recombinant human EGFR (#TP723071) was from OriGene (Rockville, MD). Recombinant human NRG1-β1 (NRG1 hereafter) (#377-HB-050) and EGF (#236-EG) ECD were provided by Biotechne R&D Systems (Minneapolis, MN). Control IgG from pooled human serum was purchased from MP Biomedicals ((#55908, Cappel; Solon, OH). Cetuximab (anti-EGFR mAb), and pertuzumab and trastuzumab (anti-HER2 mAbs) were provided by the ICM hospital pharmacy (Montpellier, France). The anti-EGFR mAb matuzumab (10) was kindly donated by Merck AG (Darmstadt, Germany). For western blotting, primary rabbit monoclonal antibodies against EGFR (#2232S), HER2 (#2242S), HER3 (#12708S), AXL (#8661), GAPDH (#14C10) and β-tubulin (#2148) were from Cell Signaling Technology (Danvers, MA). The secondary peroxidase-conjugated anti-human Fc (#A0170) and anti-His Tag (#A7058) for ELISA binding assays, and anti-rabbit IgG (#A0545) for western blotting were purchased from Sigma (Saint Louis, MO). For western blotting revealed by fluorescence detection, the secondary IRDye800-conjugated goat anti-rabbit IgG (#926-32211) and IRDye680-conjugated goat anti-mouse IgG (#926-68020) were from LI-COR Biosciences (Lincoln, NE). Immunochemistry (IHC) analysis of formalin-fixed paraffin-embedded (FFPE) sections was performed using primary antibodies against human IgG (γ-chain specific, #A0423; Dako, Glostrup, Denmark), Ki67 (#Ab254253, clone BMI1; Abcam, Cambridge, UK) and CD31 (#Ab28364; Abcam), and a secondary rabbit anti-rat antibody (IgG H+L, #31219; ThermoFisher). Immunofluorescence analysis of FFPE sections was done with primary antibodies against EGFR (Confirm anti-EGFR antibody, clone 5B7; Roche Diagnostics; Basel, Switzerland), HER2 (Pathway anti-HER2 antibody; clone 4B5; Roche Diagnostics) and HER3 (#DAK-H3-IC; Dako), and a secondary rabbit anti-mouse antibody (pan IgG_1,2,3_, #Ab133469; Abcam). Flow cytometry analysis of ADCC and CDC was performed with APC-AF750-conjugated anti-CD16 (#B49184; clone 3G8), ECD-conjugated anti-CD56 (#B49214; clone N901), and phycoerythrin-conjugated anti-IFNγ (#IM2717U; clone 45.15) antibodies from Beckman Coulter (Brea, CA), and PerCy5-conjugated anti-CD107a (#555802) and fluorescein-conjugated anti-complement fraction C4c (#F0169) antibodies from BD Biosciences (Franklin Lakes, NJ) and Dako (Agilent Technologies France, Les Ullis), respectively. The GolgiPlug reagent containing brefeldin (#555028) was from BD Biosciences, and staurosporine (#6942) and DMSO (#2438) were from Sigma. The MEK inhibitor selumetinib and the PI3K inhibitor were from Selleck Chemicals (Hoston, TX). NK cells were isolated for immunophenotyping using phycoerythrin-conjugated anti-CD3 (#555275; BD Biosciences), and APC-Cy7-conjugated anti-CD19 (#557655; BD Biosciences) antibodies for negative selection, and AF700-conjugated anti-CD45 (#560510; BD Biosciences), APC-conjugated anti-CD49b (#108910; Biolegend, San Diego, CA), fluorescein-conjugated anti-NKp46 (#137606; Biolegend), BV786 conjugated anti-CD107a (#564349; BD Biosciences), and BV421-conjugated anti-IFNγ (#505830; Biolegend) antibodies for positive selection.

### Production, purification and characterization of recombinant mono- and bi-specific antibodies

**Figure 1:**
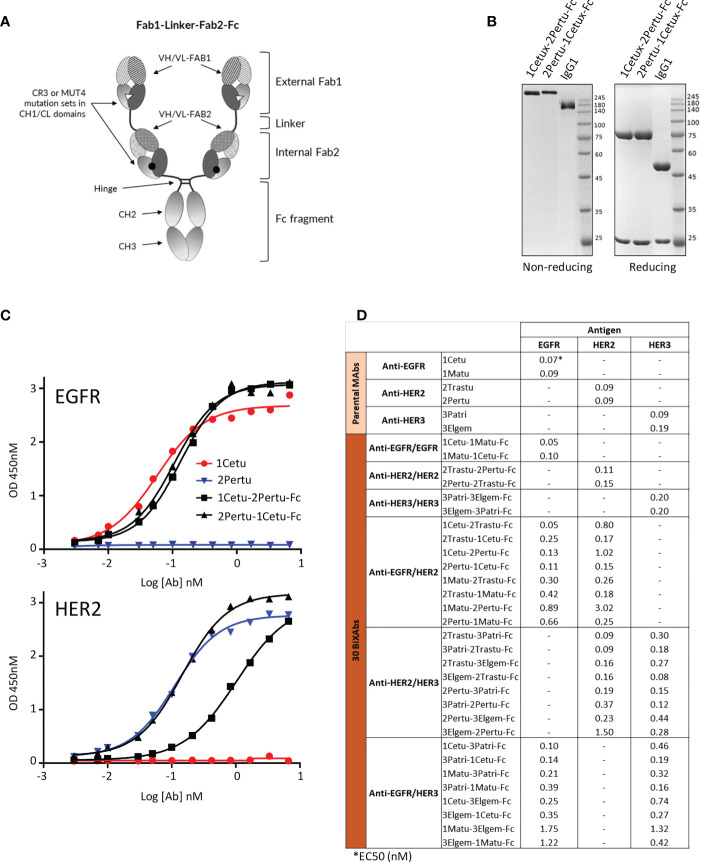
Biochemical characterization of ErbB-specific BiXAb™. **(A)** Schematic illustration of the BiXAb™ platform. **(B)** Representative SDS-PAGE analysis of the BiXAb™ 1Cetu-2Pertu-Fc and 2Pertu-1Cetu-Fc in non-reducing and reducing conditions. Proteins were stained with Coomassie brilliant blue. IgG1 was used as control and molecular weight markers are indicated. Other BiXAb™ are in **Supplementary Figure 1**. **(C)** Representative results of ELISA to assess binding of 1Cetu-2Pertu-Fc, 2Pertu-1Cetu-Fc, 1Cetu, and 2Pertu to immobilized EGFR (top panel) and HER2 (bottom panel). See **Supplementary Figure 2** for all antibodies used in this study. **(D)** EC50 values extracted from ELISA binding curves.

The variable sequences of matuzumab (1Matu), cetuximab (1Cetux), trastuzumab (2Trastu), pertuzumab (2Pertu), patritumab (3Patri; anti-HER3 mAb) and elgemtumab (3Elgem; anti-HER3 mAb) were extracted from the IMGT database (Montpellier, France). The bispecific and tetravalent BiXAb™ platform was developed by Biomunex Pharmaceuticals (patents WO2018178101 and WO2018127608), based on the initial BsAb format described by Golay et al. (61) and the accompanying patent (WO2013005194). The BiXAb™ architecture (**Figure 1A**) consists of a tetravalent IgG-like format with two Fabs arranged in tandem and joined by a novel semi-rigid pseudo-hinge linker. This linker is a human sequence that contains the IgG1 hinge, the CH2 domain N-terminus and a human Ig alpha hinge sequence. It links the CH1 domain C-terminus of the external Fab1 with the VH domain N-terminus of the internal Fab2 (WO2018127608). Moreover, an original set of mutations have been introduced at the interface of the CH1 and CL domains of the two Fabs to facilitate cognate pairing of heavy/light chains and prevent their mispairing (described in WO2018178101). The two tandem Fabs are constructed on an Fc fragment to endow the obtained BiXAb™ with immune-related mechanisms and adopt the full bispecific, bivalent antibody architecture to permit efficient binding to the two targeted epitopes. The variable regions of six mAbs were cloned in separate vectors resulting in 30 heavy chain cassettes that comprised all possible combinations of the six antibodies in both orientations, and the six light chains. The BiXAb™ synthesis required the production of one continuous fused heavy chain with two separate light chains (submitted patent; application number EP22305242.4). The BiXAb™ antibodies were produced by transient co-transfection of three genes coded on separate pQMCF vectors in a 2:1:1 (heavy chain:light chain1:light chain2) molecular ratio.

The cloning, production and purification of mAbs and BiXAb™ was performed at Icosagen (Tartu, Estonia). The thirty 250-kDa BiXAb™, derived from 1Cetu (EGF-competitive, specific for EGFR domain 3), 1Matu (EGF-non-competitive, specific for EGFR domain 3), 2Pertu (HER2 domain 2-specific; no ligand identified), 2Trastu (HER2 domain 4-specific; no ligand identified), 3Patri (NRG1-competitive and specific for HER3 domain 2) and 3Elgem (NRG1-non-competitive and specific for HER3 domains 2/4), were produced in CHO cells and purified by Protein A affinity chromatography. The control BiXAb™ HLA-DR/CD5 was described elsewhere (61) and the control CD3/CD19 BiXAb™ was provided by Biomunex Pharmaceuticals. For the quality control of the purified BiXAb™, aggregation, monomer and dimer content in native conditions were determined by size exclusion chromatography using HiLoad^®^ 26/600 Superdex^®^ 200 pg columns (Cytiva, Germany; #28-9893-36) and by assessing their thermal stability by differential scanning colorimetry. Antibodies were stored at 4°C in PBS buffer, pH 7.5.

### Electrophoresis, Coomassie blue staining, and western blotting

BxPC3 and Sw1990 cells at 80% confluence were seeded in flat-bottom plates or culture flasks (Starsted) with complete medium at 37°C for 24 h before serum starvation (culture medium with 2% FCS) for 24 h. After medium removal, tumor cells were incubated with 330 nM of BiXAb™ or 1MAbs, or a 330 + 330 nM-concentration of 2MAbs diluted in culture medium/2% FCS for 6 h. Then, cells were washed twice with cold PBS, lysed in lysis buffer (0.05 M Hepes pH 7.7, 10% glycerol, 0.15 M NaCl, 1% Triton X-100, 1 mM EDTA, 1 mM EGTA) supplemented with 1/100 solution of anti-phosphatase and protease cocktail (#7843; ThermoFisher, Waltham, MA) at 4°C for 30 min. After centrifugation at 13,000 g for 15 min, resuspension in 1X Laemmli buffer (60 mM Tris pH 6.8, 10% glycerol, 1% SDS), and heating at 95°C for 5 min, 100 µg of proteins were loaded on 7-12% electrophoresis gels and were separated at 100 V for 2 h. After liquid transfer at cold temperature and constant voltage (100 V) for 2 h, PVDF membranes were saturated in PBS/0.1% Tween (PBS-T) supplemented with 5% skimmed milk at room temperature for 2 h. Then, membranes were probed with primary antibodies diluted in PBS-T/3% BSA/0.002% sodium azide at 4°C for 18 h. Membranes were then incubated with a peroxidase-conjugated anti-rabbit antibody (1/2,000 dilution in PBS-T) at room temperature for 1 h. For enzymatic detection, western blots were revealed with the Western Lightning Ultra reagent (Perkin Elmer, Waltham, MA) and images were acquired with G:BOX (Syngene, Cambridge, UK) before quantification with the ImageJ™ software.

For Coomassie blue staining of the produced antibodies, 2 µg of protein samples were diluted in Laemmli buffer, with or without mercaptoethanol, and with or without heating at 95°C for 5 min. Then, samples were separated on 8%-12% electrophoresis gels. After 2 h-migration at 100 V, gels were rinsed once with distilled water and stained with Coomassie blue (#B0149, Sigma) under agitation at room temperature for 1 h. Then, gels were washed with acetic acid/ethanol until only the protein bands and molecular weight markers (#00193837; Lonza, Basel, Switzerland) remained visible.

### Direct ELISA binding assay and BiXAb™ EC_50_ to EGFR, HER2, or HER3

Nunc Maxisorp 96-well plates (Rochester, NY) were coated (50 µl/well) with His-tagged EGFR, HER2 or HER3 (800 ng/ml in PBS) at 4°C for 18 h. After four washes in PBS-T, plates were saturated with PBS-T/1% BSA at 37°C for 1 h. Then, two-fold or ten-fold dilutions of BiXAb™ or 1MAbs were added (final volume of 50 µl/well in PBS buffer) and incubated at 37°C for 1 h. After four washes in PBS-T, the peroxidase-conjugated anti-human Fc antibody was added (1/40000 dilution in PBS buffer) at 37°C for 1 h. Then, plates were rinsed four times in PBS-T, and 50 µl of TetraMethylBenzidine (TMB) (#34028; ThermoFisher) was added to each well. After, 10-30 min incubation at room temperature in the dark, the reaction was stopped by adding 1 M hydrochloric acid (50 µl/well) and the optical density was read at 450 nm.

### Analysis of EGFR/HER2, EGFR/HER3 and HER2/HER3 bi-specificity by sandwich ELISA

Nunc Maxisorp 96-well plates were coated with tag-free EGFR, HER2 or HER3 (1000 ng/ml in PBS buffer) (50 µl/well) at 4°C for 18 h. After four washes in PBS-T, plates were saturated with PBS-T/1% BSA at 37°C for 1 h. After washing in PBS-T, the BiXAb™ were added (various dilutions in PBS buffer) in a final volume of 50 µl/well and left at 37°C for 1 h. After four washes in PBS-T, 1000 ng/ml of His-tagged EGFR, HER2, or HER3 in PBS (50 µl/well) was added at 37°C for 1 h. Then, plates were washed in PBS-T four times before adding the peroxidase-conjugated anti-His tag antibody (1/2000 dilution in PBS buffer) (50 µl/well) at 37°C for 1 h. After four washes in PBS-T, 50 µl/well of TMB was added at room temperature in the dark. After for 10-30 min, the reaction was stopped by adding 1 M hydrochloric acid (50 µl/well) and the optical density was read at 450 nm.

### Quantification of AKT and ERK phosphorylation by time-resolved fluorescence energy transfer

ERK and AKT phosphorylation levels were quantified by time-resolved fluorescence energy transfer (TR-FRET) using the pERK Thr202/Tyr204 (#64ERKPEG) and pAKT Ser473 (#64AKSPET) TR-FRET kits (CisBio Perkin Elmer, Le Codolet, France). Fifty thousand BxPC3, AsPC-1, CFPAC, Sw1990, SKBR3 and C-PDX P4604 cells/well were grown in complete medium in sterile 96-well flat-bottom plates (#83.3924; Sarstedt, Germany) at 37°C for 24 h before serum starvation in culture medium/2% FCS for 24 h. After medium removal, cells were incubated with 70 nM of BiXAb™ or parental 1MAbs in culture medium/2% FCS (100 μl/well) for 20 min, and then 16.6 nM EGF (100 ng/ml) and 3.71 nM NRG1 (100 ng/ml) were added at 37°C for 10 min. After removal of excess antibodies and ligands by extensive washings, cells were lysed under agitation in the supplemental lysis buffer (CisBio Perkin Elmer) for 1 h. Lysates were transferred to white 384-well plates. Anti-pERK-cryptate/anti-pERK-d2 or anti-pAKT cryptate/anti-pAKT-d2 antibody pairs were added to each well and left in the dark at room temperature for 4 h, according to the manufacturer’s instructions. The TR-FRET signal (665 nm/620 nm emission ratio) was measured on a Pherastar (BMG Labtech, Ortenberg, Germany) reader and normalized to 100% binding (obtained by incubating tumor cells with the EGF+NRG1 mixture without antibodies). Negative control wells contained unstimulated/non-treated cells and labeled antibodies. Experiments were done in triplicate.

### Cell viability

Cell viability was measured with the CellTiter 96 Aqueous One Solution Cell Proliferation Assay (#G3581; Promega, Madison, WI). Three- to five-thousand BxPC3, Sw1990, and BxPC3-GR cells were seeded in complete medium in sterile 96-well flat-bottom plates (100 μl/well) (Starsted) at 37°C for 24 h before starvation (culture medium/2% FCS) for 24 h. After medium removal, cells were incubated with 330 nM BiXAb™ or 1MAbs, or 330 + 330 nM 2MAbs diluted in culture medium/2% FCS (100 µl/well) for 5 days. Cell viability (mitochondrial activity) was measured according to the manufacturer’s protocol. Plates were incubated at 37°C for 1 h to 3 h and colorimetry was measured at 490 nM with a Multiskan reader (ThermoFisher). The percentage of cell viability was normalized to medium-treated cells (i.e. 100% viability) and to DMSO-treated cells for 2 h (i.e. 0% cell viability). Experiments were done in triplicate.

### Flow cytometry analysis of the CD16-dependent NK92 cell responses for ADCC initiation (CD107a degranulation and IFNγ production)

Target (T) BxPC3, Sw1990, AsPC-1 and CFPAC cells (1x10^5^) were co-cultured with huCD16a-transfected NK92 effector (E) cells (60) at an E to T ratio of 1:2, in 200 µl of RPMI medium. Suspended cells (E+T) were co-incubated at 37°C with a saturating concentration of BiXAb™ (40 nM) for the initial screening of the 30 BiXAb™, or with a saturating concentration of BiXAb™ (40 nM), parental 1MAbs (66 nM), or 2MAbs (33 nM+33 nM) for the comparison of antibody treatments, and with the GolgiPlug reagent that contains brefeldin A to block protein transport, and with (if not in the reagent) the PerCy5-conjugated anti-CD107a antibody (1/20 dilution) to monitor degranulation of activated CD16^+^ NK92 cells. After 4 h of incubation, cells were washed, and incubated with APC-AF750-conjugated anti-CD16 (1/100 dilution) and ECD-conjugated anti-CD56 (1/25 dilution) antibodies (membrane CD16/CD56 co-labeling) at 4°C for 30 min to distinguish CD16^+^ NK92 cells from tumor cells during flow cytometry analysis. After cell fixation and permeabilization according to the supplier’s recommendations (#555028, BD Biosciences), intracellular IFNγ production by CD16^+^ NK92 cells was detected by incubation with a phycoerythrin-conjugated anti-IFNγ antibody (1/10 dilution) at 4°C for 30 min. After extensive washing, 10,000 events were acquired using a Gallios flow cytometer (Beckman Coulter). The percentages of activated CD16^+^ NK92 cells that degranulated (CD107a^+^) or produced IFNγ (IFNγ^+^) and of cells that exerted both responses (CD107a^+^IFNγ^+^) were compared by data analysis with the Kaluza software (version 2.1; Beckman Coulter).

### Flow cytometry analysis of CDC initiation by expression of the C4c complement fraction

After trypsin detachment, suspended BxPC3 cells (1x10^5^) were incubated at 37°C with a 10 μg/ml-saturating concentration of BiXAb™, parental 1MAbs or 2MAbs in culture medium/1% complete human serum (#ICSER-25ml; Serum Innovative Research, Novi, MI) for 30 min. C4c complement fraction binding was revealed by labeling cells with a FITC-conjugated anti-C4c antibody (1/100 dilution) at 4°C for 30 min. After washing, 10,000 events were acquired using a Gallios flow cytometer. The mean fluorescence intensity (MFI) was quantified with the Kaluza software.

### Measurement of caspase 3/7 activation

Caspase 3/7 activation was measured using the Caspase-Glo 3/7 assay kit (#G8091; Promega) according to the manufacturer’s protocol. Three thousand BxPC3 cells were cultured in 100 µl of complete medium/10% FCS in 96-well flat-bottom plates (Starsted) for 24 h, before serum starvation in culture medium/2% FCS for 24 h. After medium removal, cells were incubated with 330 nM 1MAbs, BiXAb™, or 2MAbs for 6 h or 24 h. Some cells were incubated with staurosporine (200 nM; positive control for caspase 3/7 activation) at 37°C for 3 h. The Caspase-Glo 3/7 reagent, in which the luminogenic caspase 3/7 substrate is mixed with the appropriate lysis buffer, was added to each well for 30 min to 3 h. Caspase 3/7 activity was correlated with luciferase intensity recorded with a Pherastar plate reader (BMG LabTech).

### Flow cytometry analysis of apoptosis

Cell apoptosis was assessed using the Annexin V/7-AAD kit (#IM3614; Beckman Coulter) according to the manufacturer’s protocol. 100,000 BxPC3, Sw1990, and C-PDX P4604 cells were grown in 1 ml of medium/10% FCS in 12-well plates (Sarstedt) at 37°C for 24 h, before serum starvation in culture medium/2% FCS for 24 h. After medium removal, cells were incubated with various concentrations of BiXAb™ or 2MAbs in medium/2% FCS at 37°C for 48 h. Some cells were incubated with staurosporine (200 nM; positive control for apoptosis) at 37°C for 3 h. After centrifugation, the cell pellet of each well was labeled with 10 µl of annexin-FITC and 20 µl of 7-AAD. After 15 min incubation at 4°C in the dark, Annexin V/7-AAD labeling was measured with a Gallios flow cytometer and analyzed with the Kaluza software.

### ELISA quantification of RTK expression

RTK expression was evaluated with a sandwich ELISA using the PathScan^®^ Cell Signaling Array kits to quantify total EGFR (#7250), HER2 (#7310C) and HER3 (#7888C). 250,000 BxPC3, Sw1990, and P4604 C-PDX cells were grown in 5 ml of complete medium/10% FCS in BP60 tissue culture dishes (#83.3901; Sarstedt) at 37°C for 24 h, before serum starvation in medium/2% FCS for 24 h. After medium removal, cells were incubated with 330 nM BiXAb™ or 1MAbs, or 330 + 330 nM of 2MAbs, diluted in 4 ml of culture medium/2% FCS, at 37°C for 6 h. Some cells were incubated with EGF (16.6 nM) plus NRG1 (3.71 nM) for 6 h, as positive control. After washings in cold PBS and cell lysis with the kit lysis reagent, EGFR, HER2 and HER3 sandwich ELISAs were performed according to the manufacturer’s protocols. Total EGFR, HER2 and HER3 protein levels were measured with a Pherastar plate reader at 450 nm.

### Phosphoproteomic profiling of RTKs and signaling kinases

The phosphoproteomic analysis of RTKs and downstream signaling kinases was performed using the human phospho-RTK (49 targets, #ARY001B), and phospho-kinase (43 targets for#ARY003B or 37 targets for ARY003C) array kits from Biotechne RD Systems. One million BxPC3, Sw1990, and C-PDX P4604 cells were grown in 7 ml of culture medium/10% FCS in BP100 tissue culture dishes (#83.3902; Sarstedt) at 37°C for 24 h, before serum starvation in medium/2% FCS for 24 h. After medium removal, cells were incubated with 70 nM BiXAb™ or 1MAbs, or 70 nM + 70 nM 2MAbs (to obtain Fab equimolarity), diluted in 6 ml of culture medium/2% FCS, at 37°C for 20 min. Then, the ligand mixture (16.6 nM EGF plus 3.71 nM NRG1) was added at 37°C for 10 min. After removing excess antibodies and ligands by washings with cold PBS, cells were lysed according to the manufacturer’s protocol, and proteins (300 µg of per condition) were loaded on spot membranes. Incubation and revelation of spot membranes were performed with the reagents included in the kits and according to the manufacturer’s protocols. Spot images were acquired with a G:BOX apparatus (Syngene), and phosphoproteomic analysis and quantification were performed with the GeneTools software (Syngene).

### Xenografts of Sw1990 and PDX P2846 pancreatic cancer cells

All *in vivo* experiments were performed in compliance with the French regulations and ethical guidelines for experimental animal studies in an accredited establishment (Agreement No. C34-172-27). Sw1990 cells (3x10^6^ cells per mouse) or the PDX P2846 at passage 8 (P8) since surgery (125 mm^3^ tumor piece per mouse) were injected subcutaneously in the right flank, or transplanted intrascapularly in the brown fat, respectively, of 6 week/old female Swiss nude (Crl : NU(Ico)-Foxn1nu) mice from Charles River (Wilmington, MA). Tumor-bearing mice were randomized in the different treatment groups (15 animals/group) when tumors reached a minimum volume of 100-150 mm^3^ for Sw1990 cell xenografts and 200 mm^3^ for P2846 xenografts. Tumor size was measured with a caliper, and volumes were calculated with the formula: D_1_ x D_2_ x D_3_/2. During the initial *in vivo* experiments, mice were treated with the three selected lead BiXAb™ 3Patri-1Cetu-Fc, 3Patri-1Matu-Fc, and 3Patri-2Trastu-Fc (17 mg/kg) and the control CD3/CD19 BiXAb™ (BiXAb™ IRR; 17 mg/kg) by intraperitoneal (ip) injection twice per week for 4 weeks (Q3D-4W). A second set of experiments was performed with the lead BiXAb™ 3Patri-1Cetu-Fc at Fc (17 mg/kg) or Fab (8.5 mg/kg) equimolar dose, the 3Patri (5mg/kg) + 1Cetu (5mg/kg) combination, the 1MAbs alone (3Patri or 1Cetu; 10mg/kg), and the control CD3/CD19 BiXAb™ (17mg/kg). For survival comparison, mice (10 animals/group) were sacrificed when tumor reached a volume of 1500 mm^3^. For *in vivo* immunophenotyping and IHC analysis, mice (5 animals/group) were sacrificed after 15 days of treatment (Q3D-2W) when tumor volume was ~250-500 mm^3^.

### Immunofluorescence and IHC analyses of tumor xenografts from BiXAb™ -treated mice (Ki67, CD31, BiXAb™ penetration)

Surgically excised Sw1990 cell and P2846 xenografts were fixed in 10% neutral buffered formalin for 24 h, dehydrated, and embedded in paraffin. Paraffin-embedded tissues were cut in 3-µm-thick sections, mounted on slides, dried at 37°C overnight, deparaffinized in xylene, and rehydrated in graded alcohols. Immunofluorescence was performed according to the manufacturer’s instructions using the Discovery Ultra autostainer (Ventana/Roche Diagnostics). For HER3 immunofluorescence analysis, antigen retrieval was performed with EnVision^®^ Target Retrieval Solution High pH (#GV804, Dako) at 97°C for 20 min, followed by incubation of tissue sections at 37°C with the mouse monoclonal anti-HER3 antibody (1/25 dilution) for 1 h. The antigen-antibody reaction was revealed using a rabbit anti-mouse pan IgG_1,2,3_ as the secondary antibody, and the Discovery Rabbit HQ and Cy5 kits (Roche) in a Discovery Ultra autostainer (Roche). Immunofluorescence signals were analyzed with a THUNDER Imager (Leica Microsystems; Wetzlar, Germany). For IHC analyses, following deparaffination, antigen retrieval was performed by incubation with the Discovery CC1 buffer (Roche) at 95°C for 24, 40, and 60 min for human IgG, Ki67 and CD31 detection, respectively. Slides were incubated at 37°C with primary antibodies against human IgG (1/2000 dilution), Ki67 (1/500 dilution) for 60 min, and with the primary antibody against CD31 (1/75 dilution) for 32 min. Signal enhancement was performed with the Discovery DAB Rabbit OmniMap Kit (Roche) for Ki67 and with the Discovery DAB Rabbit HQ Kit (Roche) for CD31 and human IgG. Slides were counterstained with hematoxylin for 8 min and manually dehydrated before mounting. Images were acquired with a Hamamatsu NanoZoomer 2.0-HT scanner at the MRI facility, and images were visualized with the NDP.view 1.2.47 software.

### NK cell immunophenotyping in BiXAb™-treated tumor xenografts

Xenografts from BiXAb™-treated mice (n=5/group) were excised one day after the last injection (Q3D-2W schedule) in 1 mM EDTA-dissociating PBS buffer. Dissociation was performed using the Tumor Dissociation Kit (#130095929; Miltenyi Biotec, Bergisch Gladbach, Germany) and the gentleMACS Octo Dissociator (Miltenyi Biotec) according to the Miltenyi protocol for dense tumors. One million of freshly-dissociated tumor cells were resuspended in 200 µl of FACS buffer (PBS, 2% FCS, 0.02% sodium azide, and 0.02% 1 mM EDTA). Each sample was pre-incubated with 50 µl of mouse FcR Blocking Reagent (#130092575; Miltenyi Biotec) at 4°C for 1 h. After washings, cells were incubated with a multicolor antibody panel containing AF700-conjugated anti-CD45 (1/400 dilution), Cy7-conjugated anti-CD19 (1/200 dilution), PE-conjugated anti-CD3 (1/400 dilution), FITC-conjugated anti-NKp46 (1/200 dilution), APC-conjugated anti-CD49b (1/200 dilution), and BV786-conjugated antiCD107a (1/200 dilution) antibodies, diluted in FACS buffer, at 4°C for 1 h. Then, cells were fixed and permeabilized using the fixation/permeabilization kit (#00512343; ThermoFisher) at 4°C for 40 min. Intracellular IFNγ staining was then performed with the BV421-conjugated anti-IFNγ antibody (1/100 in FACS buffer). Immunophenotyping was performed with the LSRFortessa™ Cell Analyzer Flow Cytometer (BD Biosciences). NK cells in BiXAb™-treated and untreated tumors were analyzed by negative/positive selection of the CD45^+^, CD3^-^, CD19^-^, NKp46^+^, CD49b^+^, CD107a^+^ and/or IFNγ^+^ phenotype.

### Western blot analysis of RTK expression in tumors

Pieces of surgical excised xenografts were washed twice in cold PBS buffer, and then lysed with RIPA lysis buffer (50 mM Tris-HCl pH 8, 150 mM NaCl, 0.5% sodium deoxycholate, 5 mM EDTA, 0.1% SDS, 1% Triton X100) supplemented with a 1/100 solution of anti-phosphatase and -protease cocktail (#7843; ThermoFisher), in tubes containing beads (MP lysing matrix; #6913100, ThermoFisher) for 20 min, and then for 10 min (by shaking at 4°C). After centrifugation at 13,000 g for 15 min, dilution in 1X Laemmli buffer (60 mM Tris pH 6.8, 10% glycerol, 1% SDS), and heating at 95°C for 5 min, proteins (100 µg) were loaded on 4-15% pre-casted electrophoresis gels (#5671084; BioRad, Hercules, CA) and were separated at 200 V constant voltage. After transfer using the Trans-blot Turbo Transfer System at constant voltage (100 V) for 30 min, nitrocellulose membranes were saturated in PBS-T/5% skimmed milk at room temperature for 1 h. Then, membranes were probed with primary antibodies diluted in PBS-T/3% BSA/0.002% sodium azide at 4°C for 18 h, followed by fluorescent IRDye 800- or 680-conjugated anti-rabbit or anti-mouse antibodies (1/20000 dilution) in BioRad blocking buffer (#12010020) and 0.2% Tween-20 at room temperature for 1 h. Fluorescence signals were detected with the Odyssey Fc Imaging System (LI-COR Biosciences).

### Data processing and network modeling

Heatmaps were obtained with R and the ComplexHeatmap library (62). The HER/AKT/ERK signaling models were computed using the aiMeRA R library (63). To cluster the antibodies on the basis of the phenotypic response, we assigned each antibody to a feature vector that included AKT and ERK activation (i.e. phosphorylation) rates in the six cell lines (BxPC3, AsPC-1, CFPAC, Sw1990, P4604, SKBR3; 12 values in total), the sum of ADCC values measured in BxPC3, Sw1990, CFPAC, and AsPC-1 cells (this total was multiplied by 6 to balance with the other features), and the sum of the viability percentages in BxPC3, BxPC3-GR, and P4604 cells (this total was multiplied by 3 to balance with the other features). Based on these feature vectors, we used hierarchical clustering with Euclidean distance (dist function in R) and Ward D method (hclust function in R).

### Statistical analysis

For *in vitro* assays, significance of comparisons among groups was determined using ANOVA followed by the Dunnett’s test (comparison with untreated cells). Significance between groups was determined with the Student’s *t*-test. Data were analyzed with GraphPad Prism 6 (San Diego, CA). The relationship between tumor growth and treatment was analyzed using a linear mixed regression model. The fixed part of the model included the variables ‘number of days post-graft’ and ‘treatment group’; interaction terms were also evaluated. Random intercepts and random slopes were included to take into account the time effect. The model coefficients were estimated by maximum likelihood. A survival analysis was performed and the event considered was a tumor volume of 1500 mm^3^. Survival rates were estimated using the Kaplan-Meier method and survival curves were compared with the Log-rank test. Statistical significance was set at p <0.05. Statistical analyses were done with STATA 16 (Stata Corporation, College Station, TX, USA). P-values: p <0.05 (*), p <0.01 (**), p <0.001 (***), ns (not significant).

## Results

### Design of 30 BiXAb™ targeting ErbB family members

Due to its controversial impact (64), we did not include HER4 targeting when we designed BiXAb™ against ErbB family members. To cover as many configurations as possible within a reasonable number of BiXAb™, we selected two parental antibodies for each receptor that target different epitopes. We used the BiXAb™ platform (**Figure 1A**) to generate 30 tetravalent IgG1-like BiXAb™ comprising all possible combinations and orientations of the six ErbB antibodies (1Cetu and 1Matu, 2Pertu, and 2Trastu, 3Patri, 3Elgem) (**Figure 1D**). We adopted the nomenclature XMAb1-YMAb2-Fc to describe the BiXAb™ antibodies in which X and Y refer to the targeted receptor (1 for EGFR, 2 for HER2, and 3 for HER3), and MAb1 and MAb2 refer to the external Fab1 and internal Fab2 linked to the Fc portion, respectively (**Figure 1A**).

We characterized the quality and integrity of the 30 BiXAb™ by SDS-PAGE, and checked their purity and stability (**Figures 1B**, **Supplementary Figure 1**). BiXAb™ integrity was not influenced by the external or internal positioning of 1Cetu and 2Pertu (**Figure 1B**, **Supplementary Figure 1A**). Size exclusion chromatography demonstrated a major single peak of monomers and differential scanning calorimetry confirmed that all BiXAb™ possessed sufficient thermodynamic stability (**Supplementary Figures 1B, C**). We compared by ELISA the concentration-dependent binding of the 30 BiXAb™ to their cognate receptors, and found EC50 values generally within 1-3 fold of those of the parental 1Mab (range: 0.05 - 3.02 nM), (**Figures 1C, D**), **Supplementary Figure 2**). Moreover, internal positioning of 1Matu, 2Pertu and 3Elgem significantly influenced the EC50 (2-30 fold), but not of 1Cetu, 2Trastu and 3Patri (0-3 fold). However, the EC_50_ was reduced (2-15 fold) when 1Matu and 2Pertu were positioned externally. This suggested that this effect was probably not due to steric constraints of the BiXAb™ format, but rather to the properties of the individual Fabs. In general, with the exception of 1Matu and 2Pertu, the external positioning of the Fab arms had minimal, if any, impact on antigen binding. This analysis also showed that several BiXAb™ had potent EC_50_ values for both targets (1-3 fold), irrespective of the positioning.

### BiXAb™ affect AKT and ERK phosphorylation, tumor cell viability, and ADCC initiation

**Figure 2:**
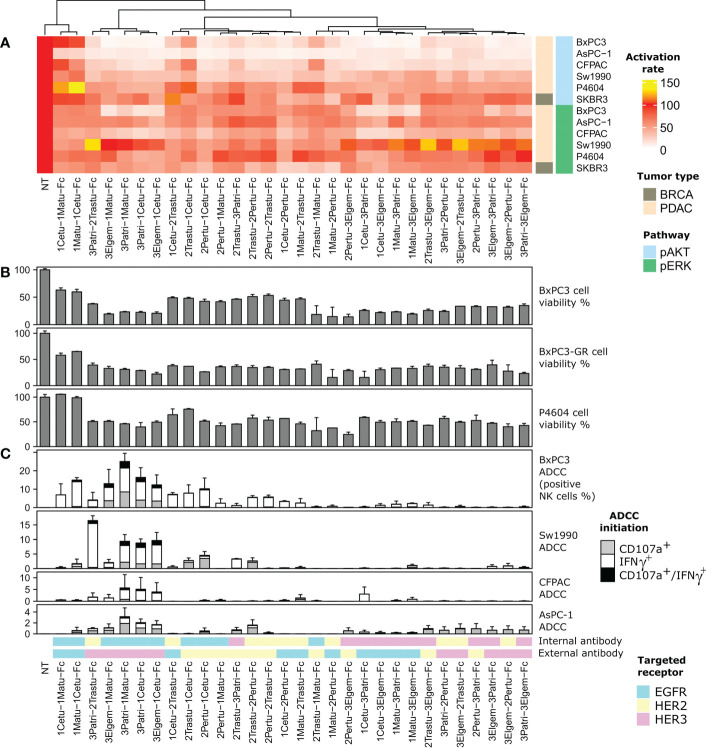
Screening of the 30 anti-ErbB BiXAb™. The dendrogram at the top represents phenotypic similarity combining, with comparable weights, the quantities reported in panels **(A, B)**, and **(C)**, as specified in the Methods section. **(A)** Inhibition of EGF/NRG1-induced phosphorylation of AKT and ERK by the 30 BiXAb™. The indicated cell lines were stimulated with BiXAb™ for 20 min before adding a mixture of NRG1 and EGF for another 10 min. The TR-FRET signal was measured relative to the maximal activation (100%; medium) obtained in NRG1/EGFR-stimulated cells without BiXAb™. **(B)** Viability of the indicated pancreatic cell lines after incubation with the 30 BiXAb™. **(C)** Antibody-Dependent Cell-mediated Cytotoxicity (ADCC) induction by BiXAb™ through CD107a degranulation and IFNγ secretion. Cells were co-incubated with CD16-transfected NK92 cells, and incubated with BiXAb™ for 4 h. The percentage of effector NK cells positive for CD107a and/or IFNγ was measured by flow cytometry. Data are the mean ± SEM of three experiments. CDC analysis is in **Supplementary Figure 3**.

AKT and ERK are key signaling hubs to transduce ErbB-mediated phosphorylation, leading to cell proliferation. EGF and NRG1 are the main ligands to induce AKT and ERK signaling as well as homo- and hetero-dimerization of ErbB receptors (65). Therefore, we tested whether our BiXAb™ could block EGF/NRG1-induced AKT and ERK phosphorylation in six tumor cell lines (**Figure 2A**, **Supplementary Table 1**). In the three pancreatic cancer cell lines (BxPC3, AsPC-1 and CFPAC), most BiXAb™ inhibited AKT phosphorylation, except 2Trastu-1Cetu-Fc and 2Trastu-1Matu-Fc (and the structural isomers). We observed moderate to low reduction of AKT phosphorylation in Sw1990, C-PDX P4604, and SKBR3 (HER2-amplified breast cancer) cell lines. The BiXAb™ 1Cetu-1Matu-Fc and 1Matu-1Cetu-Fc antibodies, which only target EGFR, had very little effect on AKT phosphorylation. ERK phosphorylation changes in pancreatic cancer cell lines were more heterogeneous. The anti-EGFR/HER3 BiXAb™ 3Patri-1Cetu-Fc, 3Patri-1Matu-Fc, and 3Elgem-1Cetu-Fc (and structural isomers) were the most efficient in inhibiting ERK phosphorylation, except in Sw1990 cells where the anti-EGFR/HER2 BiXAb™ 1Cetu-2Trastu-Fc and 1Cetu-2Pertu-Fc (and structural isomers) were the most potent. Conversely, anti-HER2/HER3 BiXAb™ increased ERK phosphorylation. In SKBR3 cells, anti-EGFR/HER2 BiXAb™ inhibited both AKT and ERK phosphorylation more efficiently than the other BiXAb™.

Most BiXAb™ inhibited cell viability by at least 50% in C-PDX P4604 cells, and in wild-type and gemcitabine-resistant (GR) BxPC3 cells (**Figure 2B**; **Supplementary Table 2**). The anti-HER2/HER3 BiXAb™ 2Pertu-3Elgem-Fc and 3Patri-2Tastu-Fc, the anti-EGFR/HER2 BiXAb™ 1Matu-2Pertu-Fc and 2Trastu-1Matu-Fc, and the anti-EGFR/HER3 BiXAb™ 3Elgem-1Cetu-Fc, 3Elgem-1Matu-Fc, 3Patri-1Cetu-Fc and 3Patri-1Matu-Fc were the most efficient in reducing cell viability.

To relate cell signaling and cell viability with immune-induced mechanisms, we investigated Fc-dependent NK cell responses (i.e. degranulation for ADCC assessment and antibody-dependent cytokine production) by dual CD107a/IFNγ labeling (**Figure 2C**; **Supplementary Table 3**), and complement cascade initiation by C4c labeling (**Supplementary Figure 3**), following incubation of BxPC3, Sw1990, CFPAC and AsPC-1 cells with different BiXAb™. Many BiXAb™ induced activation of NK cells (**Figure 2C**), but not of the classical complement cascade (**Supplementary Figure 3**), except 1Matu-1Cetu-Fc as previously reported (66). Among the 30 BiXAb™, the anti-EGFR/HER3 BiXAb™ with the Fab HER3 as Fab1 and the Fab EGFR as Fab2 were the most effective to initiate ADCC. Their efficiency progressively increased from AsPC-1 to BxPC3 cells. EGFR valence was generally required to induce ADCC, except for the anti-HER2/HER3 BiXAb™ 3Patri-2Trastu-Fc that was very efficient in Sw1990 cells. In this case, the external position of the HER3 Fab was required to induce activation. All BiXAb™ that target only one receptor showed lower ADCC activity, with the exception of 1Matu-1Cetu-Fc (and structural isomers). The best BiXAb™ for ADCC induction were 3Patri-1Matu-Fc, 3Patri-1Cetu-Fc, and 3Elgem-1Cetu-Fc.

On the basis of these results, we selected four molecules for further characterization: 2Trastu-1Cetu-Fc, 3Patri-2Trastu-Fc, 3Patri-1Cetu-Fc, and 3Patri-1Matu-Fc that target EGFR/HER2, HER2/HER3, and EGFR/HER3, respectively. These four BiXAbs™ (i) displayed EC_50_ affinity values to their cognate receptor quite similar (1-3 fold) to those of the parental 1MAbs (**Figure 1D**), (ii) were among the strongest inhibitors of EGF/NRG1-induced phosphorylation of AKT and ERK (**Figure 2A**), (iii) were among the most efficient in reducing cell viability (**Figure 2B**), and (iv) were strong ADCC inducers (**Figure 2C**) in pancreatic cancer cell lines. Moreover, previous studies already demonstrated the increased cytotoxicity of 2Trastu+1Cetu combination in pancreatic cancer models and in patients (10–12, 17).

### Bispecificity of the four selected lead BiXAb™

**Figure 3:**
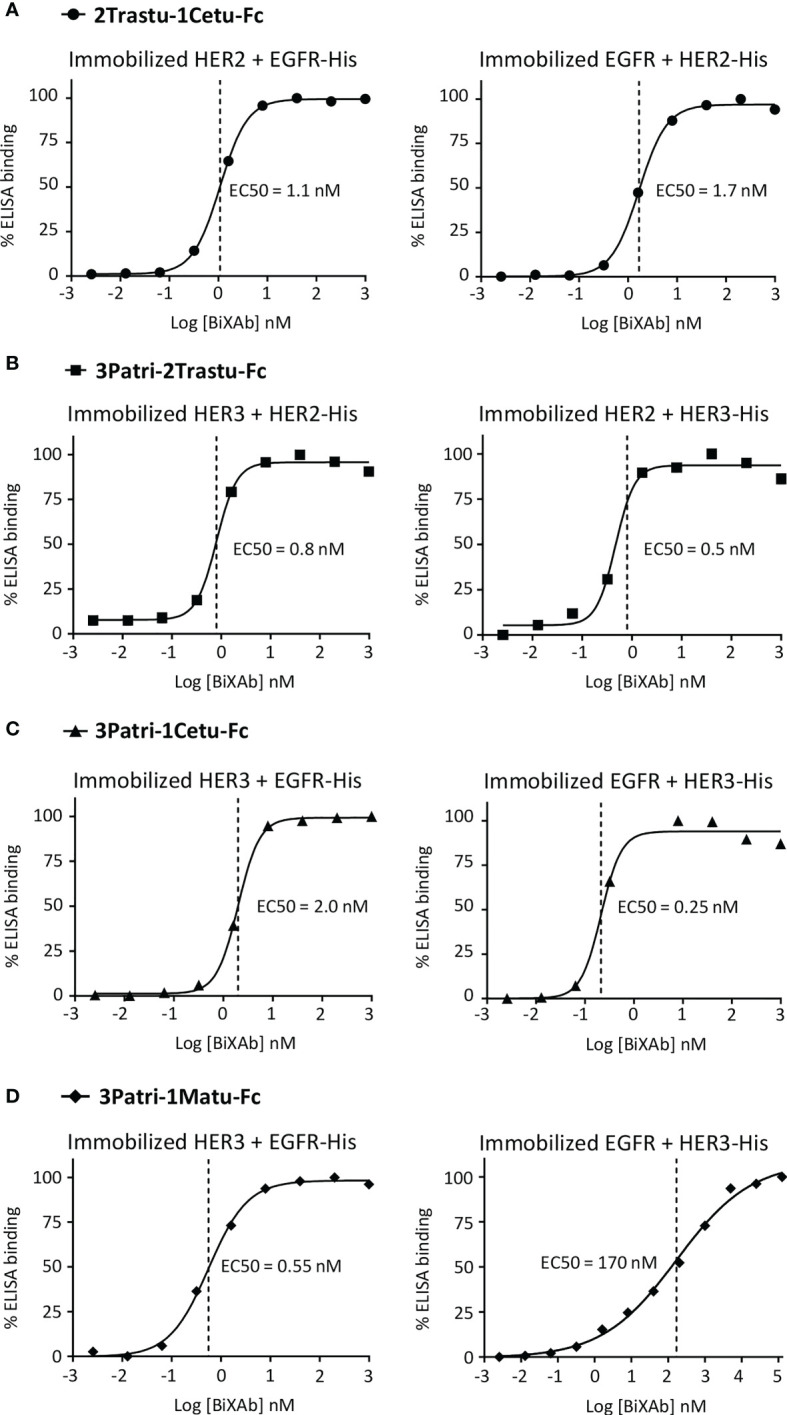
ELISA to assess the simultaneous binding of **(A)** 2Trastu-1Cetu-Fc to immobilized HER2 and EGFR-His (left) and immobilized EGFR and HER2-His (right); **(B)** 3Patri-2Trastu-Fc to immobilized HER3 and HER2-His (left) and immobilized HER2 and HER3-His (right); **(C)** 3Patri-1Cetu-Fc to immobilized HER3 and EGFR-His (left) and immobilized EGFR and HER3-His (right); and **(D)** 3Patri-1Matu-Fc to immobilized HER3 and EGFR-His (left) and immobilized EGFR and HER3-His (right). The EC50 values calculated from the ELISA binding curves are indicated.

To confirm the bispecificity of the four selected BiXAb™, we used a sandwich ELISA approach to determine whether they simultaneously co-engaged their two cognate receptors through initial engagement by the external (**Figure 3**, left panels) or internal (**Figure 3**, right panels) Fab arm, and then binding of the second free Fab arm. The BiXAb™ 2Trastu-1Cetu-Fc, 3Patri-2Trastu-Fc, and 3Patri-1Cetu-Fc showed nearly identical dose-dependent bispecific binding profiles (**Figures 3A–C**), with similar EC_50_ values regardless of the Fab engagement (between 0.25 nM and 1.7 nM). Conversely, the BiXAb™ 3Patri-1Matu-Fc displayed better bispecific binding when initially immobilized to the HER3 receptor *via* the external Fab 3Patri compared with immobilization through EGFR binding by the internal Fab 1Matu (EC_50 = _0.55 nM and 170 nM, respectively) (**Figure 3D**). This significant binding loss could be due to a greater dissociation constant of 1Matu or to steric hindrance that inhibits binding of soluble His-tagged HER3 to the external Fab 3Patri when the internal Fab 1Matu is immobilized on EGFR. These observations can be related to the results of the initial ELISA screening (**Figure 1D**, **Supplementary Figure 2**) in which EC_50_ values for EGFR binding were worse when Fab 1Matu was involved in the BiXAb™ core.

### Impact of the four lead BiXAb™ *versus* 2MAbs or 1MAbs on phosphoproteomic profiles and ErbB member expression

**Figure 4:**
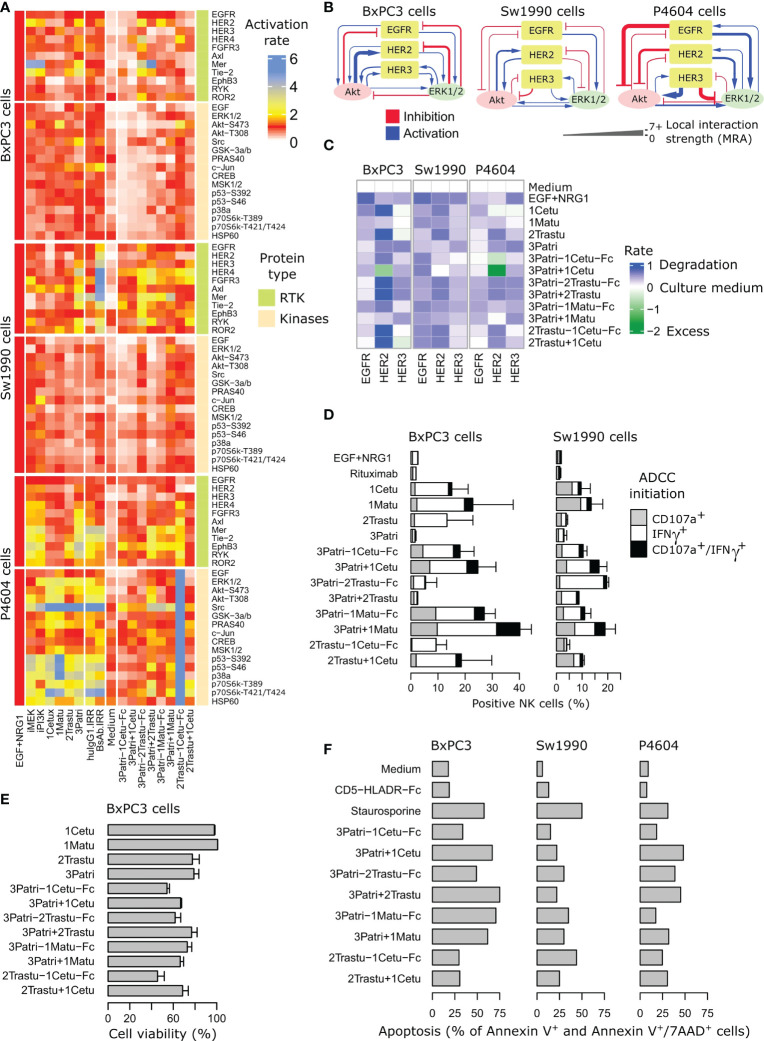
Comparison of the four lead BiXAb™ vs 2MAbs and single parental antibodies (1MAbs). **(A)** Effect on the phosphoproteome. Cells were pre-stimulated with BiXAb™, 2MAbs or 1MAbs for 20 min before adding a mixture of NRG1 and EGF for 10 min. Activation rate is relative to the maximal (100%) phosphorylation obtained in NRG1/EGFR-stimulated cells without antibodies. HuIgG1 IRR and HLA-DR-CD5-Fc were used as negative controls for 1MAbs and BiXAb™, respectively. The tyrosine kinase inhibitors iMEK and iPI3K were used for comparison. **(B)** Feedback loop strengths by modular response analysis in the indicated pancreatic cancer cell lines. **(C)** Effect on ErbB expression. The indicated cell lines were incubated with the indicated antibodies for 6 h. After cell lysis, EGFR, HER2 and HER3 levels were evaluated by sandwich ELISA (RD Systems) using receptor-specific antibodies. The percentage of expression was quantified relative to negative control (culture medium alone). The NRG1+EGF mixture was used as positive control. **(D)** Effect on ADCC assessed by monitoring CD107a degranulation and IFNγ secretion. The indicated cell lines were co-cultured with CD16-transfected NK92 cells and incubated with BiXAb™, 2MAbs or 1MAbs for 4 h. The percentage of effector NK cells positive for CD107a and/or IFNγ was measured by flow cytometry. Rituximab was used as irrelevant antibody. Data are the mean ± SEM from three replicates. The NRG1 + EGF mixture was used as negative control of ADCC. **(E)** Effect on cell viability. BxPC3 cells were incubated with BiXAb™, 2MAbs or 1MAbs for 5 days, and cell viability was measured using the MTS proliferation assay. **(F)** Effect on cell apoptosis. The indicated cells were incubated with BiXAb™ or 2MAbs for 48 h, and then labeled with fluorescein-conjugated Annexin V and 7-AminoActinomycin-D before apoptosis measurement (%) by flow cytometry. Medium and staurosporine (3 h incubation) were used as negative and positive controls of apoptosis, respectively. HLADR-CD5-Fc was used as irrelevant BiXAb™. Caspase 3/7 activation is reported in **Supplementary Figure 5**.

To compare more globally the changes triggered by the four lead BiXAbs™ and the respective 1MAbs and 2MAbs on ErbB/AKT/ERK signaling, we used two phosphoproteomic arrays that included ~90 RTKs and downstream kinases to determine the whole phosphoproteomic profiles in three pancreatic cancer cell lines. We initially measured the abundance of phosphorylated (i.e. activated) proteins after stimulation of BxPC3, Sw1990 and C-PDX P4604 cells with the ligand mixture EGF + NRG1 (**Figure 4A**, **Supplementary Figure 4**, **Supplementary Tables 4**, **5**). We hypothesized that ligand-stimulated cells reflected more accurately the tumor cell biology. We found that the phosphoproteome was more efficiently inhibited by the four BiXAb™ and 2MAbs than 1MAbs, MEK inhibitor and PI3K inhibitor. In some cases, especially in C-PDX P4604 cells, phosphorylation was increased. BxPC3 cells were the most responsive to BiXAb™ and 2MAbs, whereas Sw1990 cells and especially C-PDX P4604 cells were less affected. In all cell types, the BiXAb™ 3Patri-1Cetu-Fc displayed the strongest inhibitory effect on the phosphoproteome, notably on intracellular kinases. 2Trastu-1Cetu-Fc was the least efficient molecule. Some BiXAb™ affected the phosphoproteome profile in a different manner compared with their matching 2MAbs (*e.g.*, 3Patri-2Trastu-Fc and 3Patri-1Matu-Fc), suggesting differences in the mechanisms of action. Overall, BiXAb™ were more inhibitory than 2MAbs in approximately one third of all the phosphoproteomic experiments, showed equivalent inhibitory effects in another one third, and were less effective than 2MAbs in the last third. In this last group, we cannot rule out that the binding efficiency of one of the BiXAb™ arms might have been weakened in the BiXAb™ format.

As ErbB computational models to understand the activity of antibody compounds were previously performed by taking into account feedback loops in RTK signaling (5, 6), we then assessed the feedback loop strengths in BxPC3, Sw1990 and C-PDX P4604 cells (**Figure 4B**) by mathematical modeling using the modular response analysis methodology (63, 67). C-PDX P4604, the least responsive cell line (**Figure 4B**, see also **Supplementary Figure 4**), also harbored the most deeply rewired signaling network. This is in agreement with the known intrinsic resistance to therapy that can be caused by strong feedback loops (5). Beside changes in downstream kinase signaling, increased ErbB degradation is a well-known and important feature of treatment with antibodies against these RTKs. Data obtained in the three pancreatic cancer cell lines (**Figure 4C**) revealed decreased expression of EGFR, HER2 and HER3 (comparable profiles in all cell lines) upon incubation with BiXAb™ and 2MAbs, except for the 2MAbs 3Patri+1Cetu that led to increased HER2 expression in BxPC3 and C-PDX P4604 cells, unlike the BiXAb™ 3Patri-1Cetu-Fc. The response was more receptor-specific with 1MAbs, which was expected.

### Impact of the four lead BiXAb™, 2MAbs and 1MAbs on ADCC initiation, cell viability, and cell apoptosis

Clinically-approved antibodies against ErbB receptors act predominantly through ADCC to reduce tumor growth (56, 57). To compare the impact of the four selected BiXAbs™ versus 2MAbs and 1MAbs on ADCC initiation (NK cell activation), we incubated co-cultures of BxPC3 or Sw1990 cells and NK92 cells with 66 nM of BiXAb™, 2MAbs or 1MAbs, to maintain Fc equimolarity for all antibodies. In cultures incubated with 1MAbs, the anti-EGFR antibody induced the highest activation of NK cells: from 12 to 20% for 1Cetu and from 15% to 24% for 1Matu, in function of the pancreatic target cancer cell line (**Figure 4D**). We observed the lowest NK cell activation rate with the anti-HER2 1MAb 2Trastu, and no activation with the anti-HER3 1MAb 3Patri (levels comparable to medium alone or rituximab). 2MAbs and BiXAb™ increased ADCC initiation, especially when 3Patri was present (**Figure 4D**). 2MAbs were more effective than BiXAb™. However, 3Patri-2Trastu-Fc was the weakest ADCC inducer in BxPC3 cells (5% of activation), but the best inducer in Sw1990 cells (20% of activation), thus confirming the results of **Figure 2C**. In both cell lines, the anti-EGFR/HER3 BiXAb™ 3Patri-1Cetu-Fc and 3Patri-1Matu-Fc were the strongest ADCC inducers (10% to 28% activation), whereas 2Trastu-1Cetu-Fc was the least effective. Altogether, the BiXAb™ format did not globally improve ADCC initiation compared with 2MAbs, at least when the anti-EGFR valence was involved. We obtained similar results for complement cascade induction (**Supplementary Figure 3**), where the 1Cetu+1Matu combination induced CDC activation, whereas the related BiXAb™ 1Matu-1Cetu-Fc and 1Cetu-1Matu-Fc did not.

To further compare the four lead BiXAbs™ and the respective 1MAbs and 2MAbs, we determined whether they could inhibit viability and apoptosis of pancreatic cancer cell lines, as already demonstrated for other antibodies (10, 12, 26, 58). Three of the four lead BiXAb™ were more efficient than 2MAbs or 1MAbs to reduce BxPC3 cell viability (**Figure 4E**): ~50% of viability reduction with 3Patri-1Cetu-Fc and 2Trastu-1Cetu-Fc. Caspase 3/7 activation and apoptosis induction were comparable in BxPC3, Sw1990 and C-PDX P4604 cells incubated with BiXAb™ or 2MAbs (**Figure 4F**). The anti-EGFR/HER3 BiXAb™ 3Patri-1Cetu-Fc and 3Patri-1Matu-Fc strongly increased caspases 3/7 activation at the early time point (6h) (**Supplementary Figure 5**).

### Initial pre-clinical evaluation of three lead BiXAb™

**Figure 5:**
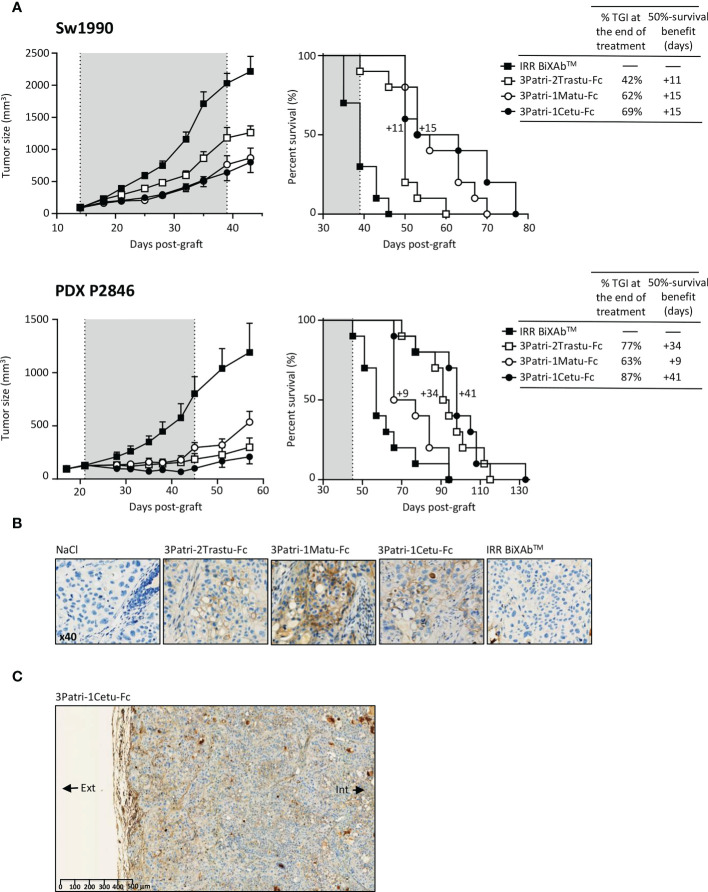
Pre-clinical evaluation of the three selected lead BiXAb™ 3Patri-2Trastu-Fc, 3Patri-1Matu-Fc, and 3Patri-1Cetu-Fc. **(A)** Tumor growth (left) and survival (middle) of BiXAb™-treated mice xenografted with Sw1990 and PDX P2846 PDAC cells. The percentage of tumor growth inhibition (TGI) at treatment end, and the 50% survival benefit (days) are indicated in the right panel. **(B)** Immunohistochemistry analysis of BiXAb™ penetration in Sw1990 cell xenografts. BiXAb™ were detected using peroxidase-conjugated anti-human Fc. IRR BiXAb™, BiXAb™ targeting HLA-DR and CD5 (negative control). NaCl was used as negative control. **(C)** Tumor penetration of 3Patri-1Cetu-Fc evaluated in whole tumor slices at lower magnification. The external (Ext) and internal (Int) parts of the tumor slice are indicated.

On the basis of the *in vitro* comparison of the four lead BiXAb™ with their 2MAbs and 1MAb counterparts, we eliminated 2Trastu-1Cetu-Fc (without HER3 valence) due to its lower potential to inhibit the phosphoproteome and to induce ADCC. We then compared the antitumor efficacy of the other three leads in mice xenografted with Sw1990 cells or the PDX P2846 (**Figure 5A**). We chose the PDX P2846 because it was from an untreated patient at surgery time. At day 39 post-Sw1990 cell xenograft (end of treatment), tumor growth inhibition was highest with 3Patri-1Cetu-Fc compared with control IRR BiXAb™ (69%; p <0.001 *vs* control), and also compared with 3Patri-1Matu-Fc (62%; p <0.001 *vs* control) and with 3Patri-2Trastu-Fc (42%; p=0.038 *vs* control). In agreement, survival (Kaplan Meier curves) was significantly increased by 15 days in mice treated with 3Patri-1Cetu-Fc- and with 3Patri-1Matu-Fc mice compared with IRR BiXAb™ (p <0.001). 3Patri-2Trastu-Fc increased survival by 11 days. At day 45 post-PDX P2846 xenograft (end of treatment), tumor growth inhibition was highest with 3Patri-1Cetu-Fc (87%; p <0.001 compared with IRR BiXAb™), followed by 3Patri-2Trastu-Fc (77%; p<0.001 *vs* control) and 3Patri-1Matu-Fc (63%; p=0.038 *vs* control). The median survival was increased by 41 days in mice treated with 3Patri-1Cetu-Fc (p <0.001 *vs* control), by 34 days in mice treated with 3Patri-2Trastu-Fc (p <0.001 *vs* control) and by 9 days in the 3Patri-1Matu-Fc group (p=0.027 *vs* control).

**Figure 6:**
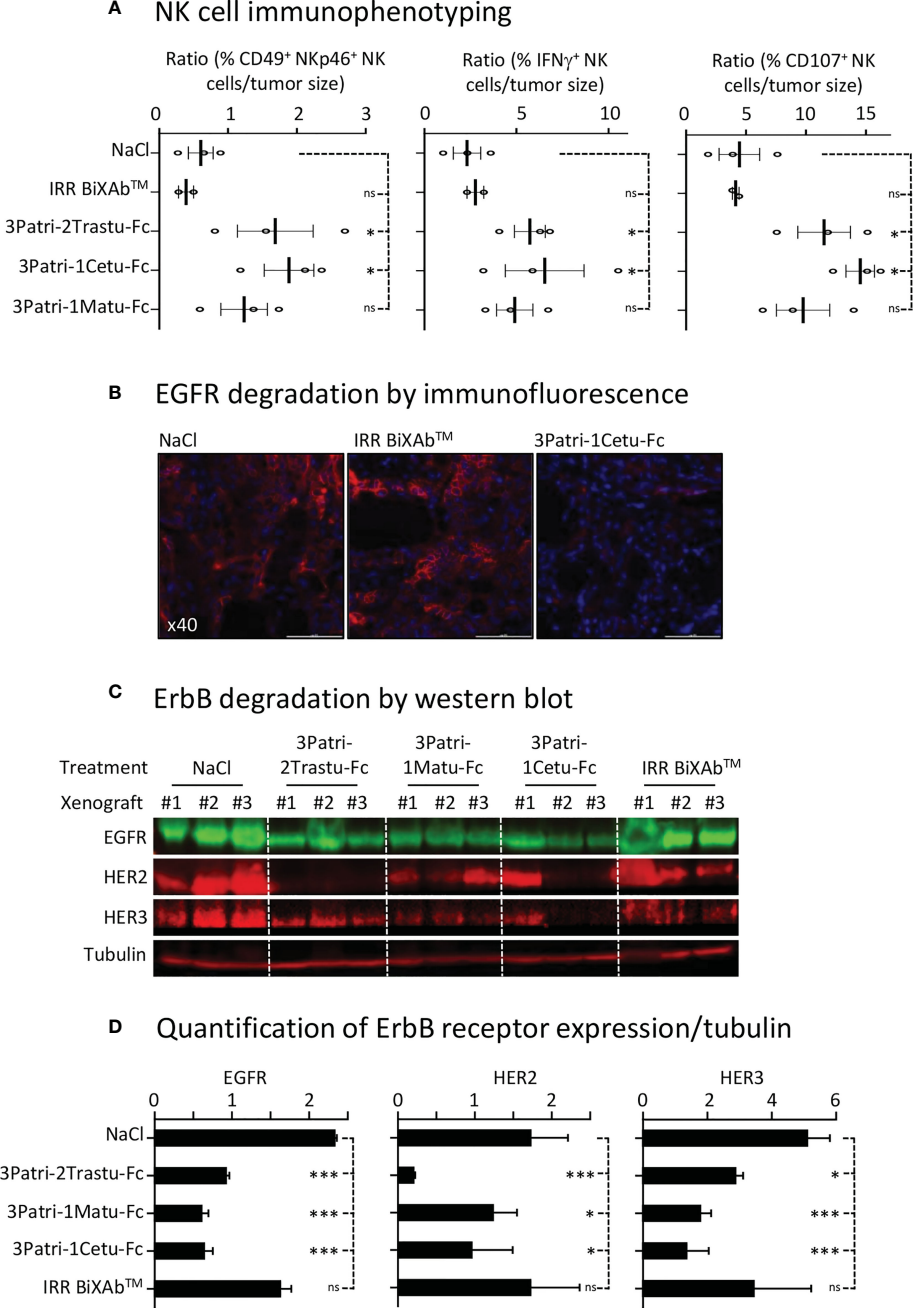
NK cell engagement and receptor expression. **(A)** Immunophenotyping by flow cytometry of NK cells in Sw1990 cell xenografts resected from BiXAb™-treated mice. Dissociated cells were labeled with human CD49-, NKp46-, IFNγ- and CD107-specific antibodies. **(B)** Immunofluorescence analysis of EGFR expression by immunofluorescence in 3Patri-1Cetu-Fc-, IRR BiXAb™- and NaCl-treated Sw1990 cell xenografts. **(C)** Western blot analysis of ErbB receptor expression in Sw1990 cell xenografts from mice treated with NaCl, 3Patri-2Trastu-Fc, 3Patri-1Matu-Fc, or 3Patri-1Cetu-Fc. Tubulin was used as loading control. **(D)** Western blots were quantified using the LI-COR Odyssey imaging system. ErbB expression level was normalized to tubulin expression level. Each experiment was performed using three Sw1990 cell xenografted mice. IRR BiXAb™, negative control. Data are the mean ± SEM. P-values: p <0.05 (*), p <0.01 (**), p <0.001 (***), ns (not significant).

We monitored BiXAb™ penetration in Sw1990 cell xenografts by IHC using an anti-human Fc antibody. BiXAb™, particularly 3Patri-1Cetu-Fc, deeply penetrated into the tumors as indicated by the outer and inner core labeling in tumor tissue cryosections (**Figures 5B, C**). We did not observe any labeling in tumor xenografts from mice treated with vehicle (NaCl) or IRR BiXAb™. Next, we immunophenotyped NK cells within the tumors and quantified ErbB receptor expression. NK cell immunophenotyping showed that treatment with ErbB-specific BiXAb™ increased the percentage of CD49^+^ NKp46^+^ NK cells that expressed IFNγ/CD107 (**Figure 6A**), in line with our initial *in vitro* ADCC results (**Figures 2C**, **4D**). The three lead BiXAb™ also decreased receptor expression in xenografts, as determined by immunofluorescence analysis of EGFR expression in 3Patri-1Cetu-Fc-treated xenografts (**Figure 6B**) and by western blotting (EGFR, HER2, HER3 expression for all treatment groups) (**Figure 6C**). Quantification of the western blot results (**Figure 6D**) showed that all three BiXAb™ similarly reduced EGFR expression. Conversely, 3Patri-2Trastu-Fc was most efficient in reducing HER2 expression, and 3Patri-1Cetu-Fc and 3Patri-1Matu-Fc in reducing HER3 expression.

### Pre-clinical evaluation of 3Patri-1Cetu-Fc, the best-in-class BiXAb™

**Figure 7:**
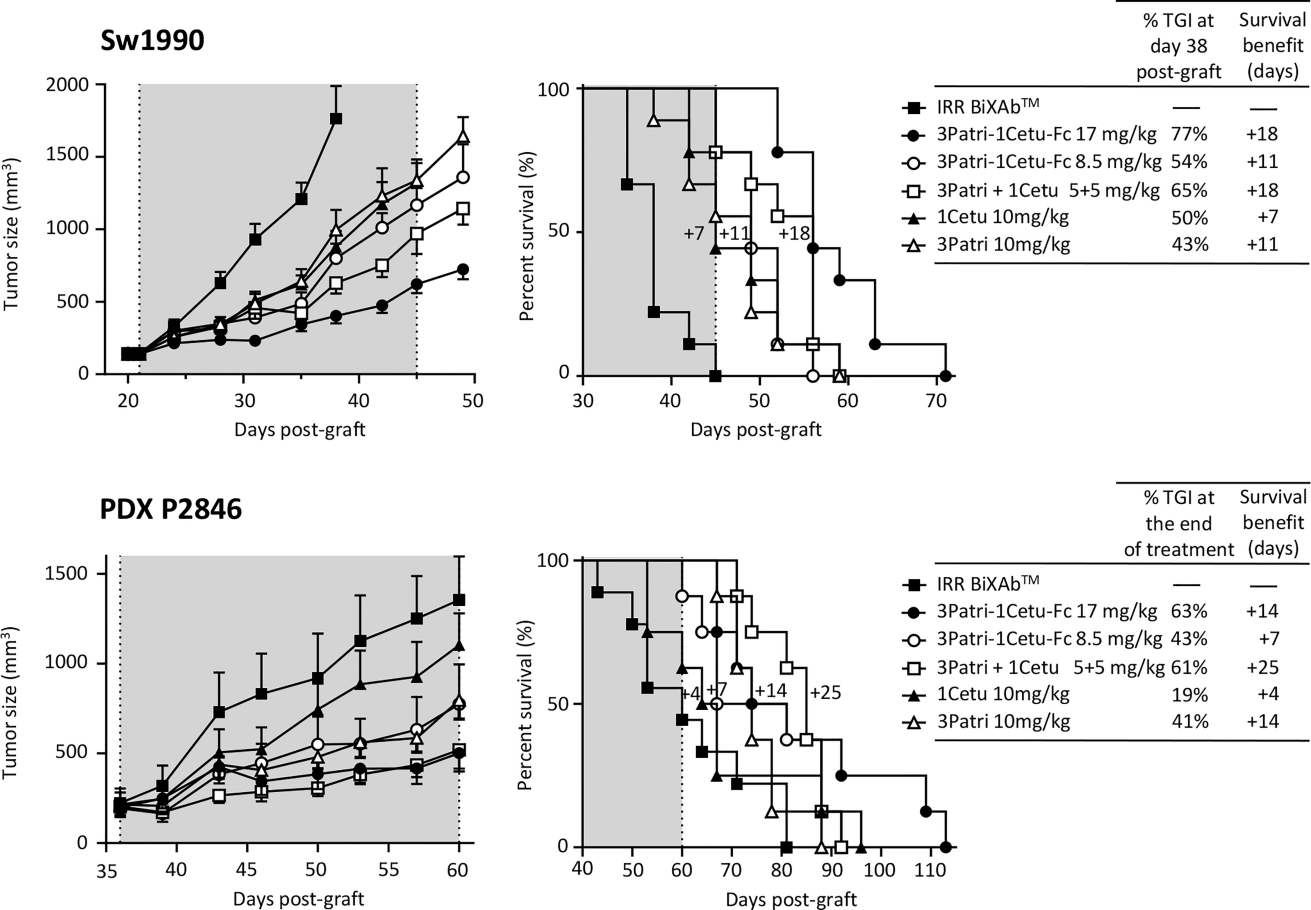
In vivo effects of the best-in-class BiXAb™ 3Patri-1Cetu-Fc versus 2MAbs and 1MAbs. Tumor growth (left panel) and survival (middle panel) of mice xenografted with Sw1990 and PDX P2846 PDAC cells treated with the indicated antibodies (n=10 mice/group). The percentage of tumor growth inhibition (TGI), and the 50% survival benefit (days) are indicated in the right panel. The BiXAb™ 3Patri-1Cetu-Fc was used at Fab equimolarity (8.5 mg/kg) or Fc equimolarity (17 mg/kg).

We selected the BiXAb™ 3Patri-1Cetu-Fc as best-in-class because it induced the strongest tumor growth inhibition and longest median survival in xenografted mice. We compared the effect on tumor growth of 3Patri-1Cetu-Fc (at equimolar doses of Fc: 17 mg/kg; or at equimolar doses of Fab: 8.5 mg/kg), of the 2MAb 3Patri+1Cetu (5 + 5 mg/kg), and of the parental 1MAbs 3Patri and 1Cetu (10 mg/kg). At day 38 post-Sw1990 cell xenograft (**Figure 7**, upper panel), tumor growth was reduced by 77% in mice treated with Fc equimolar doses of 3Patri-1Cetu-Fc (p <0.001 *vs* IRR BiXAb™), by 65% in mice treated with 1Cetu+3Patri (p <0.001), by 54% in mice treated with Fab equimolar doses of 3Patri-1Cetu-Fc (p <0.001), by 50% in mice treated with 1Cetu (p <0.001) and by 43% in mice treated with 3Patri (p <0.001). The median survival was increased by 18 days (p <0.001) in mice treated with 17 mg/kg of 3Patri-1Cetu-Fc and with 3Patri+1Cetu, by 11 days in mice treated with 8.5 mg/kg of 3Patri-1Cetu-Fc and with 3Patri, and by 7 days in mice treated with 1Cetu (**Figure 7**, upper panel). We obtained similar results in mice xenografted with the PDX P2846 (**Figure 7**, lower panel). At day 60 post-graft (treatment end), tumor growth was reduced by 63%, 61%, 43%, 41%, and 19% in mice treated with Fc equimolar doses of 3Patri-1Cetu-Fc (p <0.001 compared with IRR BiXAb™), with 2MAbs (p <0.001), with Fab equimolar doses of the BiXAb™ (p <0.001), with 3Patri (p <0.001), and with 1Cetu (p=0.001), respectively. Mouse survival was increased by 25 days in mice treated with 2MAbs (p=0.002), by 14 days in mice treated with 17 mg/kg BiXAb™ and 3Patri, by 7 days in mice treated with 8.5 mg/kg BiXAb™, and by 4 days with 1Cetu.

**Figure 8:**
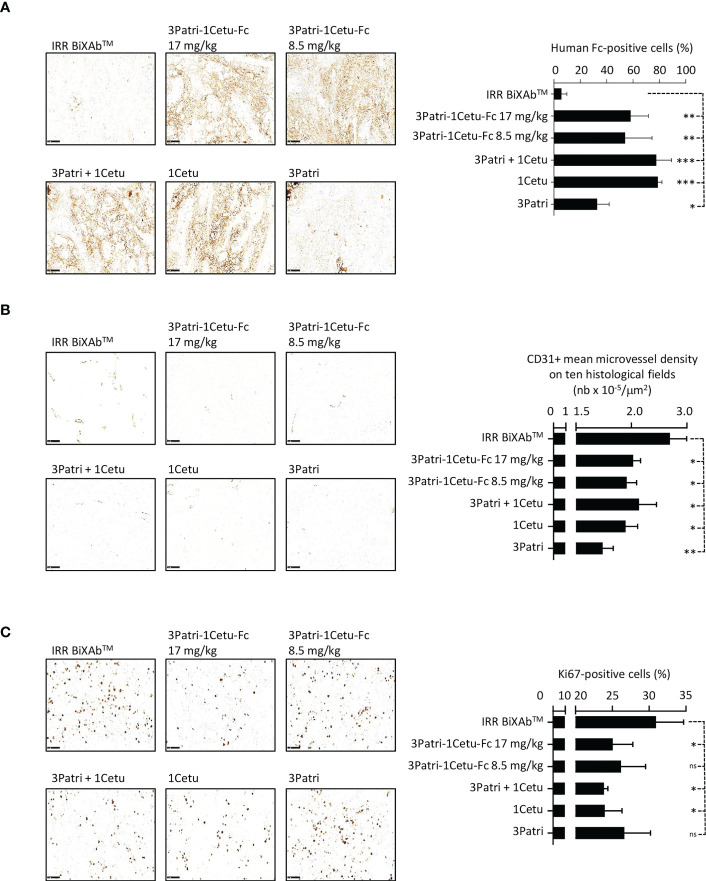
Characterization of 3Patri-1Cetu-Fc tumor penetration, and its effects on tumor angiogenesis and proliferation in mice xenografted with Sw1990 cells. **(A)** Immunohistochemistry analysis of the penetration of the BiXAb™ 3Patri-1Cetu-Fc, the 2MAbs 3Patri+1Cetu, and 3Patri and 1Cetu in tumor xenograft sections (x20 magnification; left panels). Antibodies were detected with a peroxidase-conjugated anti-human Fc. Human Fc-positive cells (right panel) were quantified in the whole tumor section using the QuPath software. Immunohistochemistry analysis (x20 magnification; left panel) of CD31 angiogenesis **(B)** and Ki67 proliferation **(C)** in Sw1990 cell xenografts. CD31+ microvessel density was estimated as the mean value in ten histological fields using the Image J software. The percentage of Ki-67+ cells in tumors was quantified in the whole tumor section using the Qupath software. IRR BiXAb™, negative control BiXAb™. Data are the mean ± SEM (n=5 mice/group). P-values: p <0.05 (*), p <0.01 (**), p <0.001 (***), ns (not significant). Immunohistochemistry analysis of P2846 xenografts is in **Supplementary Figure 6**.

The characterization of antibody penetration in Sw1990 cell xenografts and its quantification in tumor xenografts from five mice/treatment group (**Figure 8A**) showed that most antibodies strongly bound to tumor cells, except 3Patri. We observed no or low signal in tumor sections from mice treated with IRR BiXAb™ (control). Moreover, in tumor tissue samples from mice treated with the BiXAb™, 2MAbs, and 1MAbs, the density of CD31^+^ microvessels (**Figure 8B**) and the number of Ki67^+^ cells (**Figure 8C**) were reduced compared with control. We obtained similar results also in mice xenografted with the PDX P2846, except for 1Cetu (**Supplementary Figure 6**). In both models, CD31+ microvessel density was much lower in mice treated with 3Patri (against HER3) than 1Cetu (against EGFR) (**Figure 8B**, **Supplementary Figure 6A**). The number of Ki67+ proliferating cells was lowest in the 1Cetu group in the Sw1990 cell xenograft model (**Figure 8C**), but not in the PDX P2846 model where 1Cetu displayed the lowest efficacy (**Supplementary Figure 6B**). 3Patri-1Cetu-Fc at Fc equimolar doses (17 mg/kg) was the most effective to decrease EGFR and AXL expression, particularly in the PDX P2846 model (**Supplementary Figures 7A, B**). Lastly, in Sw1990 cell xenografted mice, the proportion of CD49^+^ NKp46^+^ NK cells tended to increase mainly after treatment with 17 mg/kg of 3Patri-1Cetu-Fc (**Supplementary Figure 7C**).

## Discussion

Using BiXAb™, a novel tetravalent, bispecific antibody format platform, we explored the positioning and activity of Fabs targeting the ErbB family of RTKs with the aim of identifying candidates that could potentially be used for pancreatic cancer treatment. We combined pairs of Fabs to generate BiXAb™ and assessed them in a screening funnel with functional (receptor binding and degradation, cell signaling and growth inhibition, apoptosis induction) and immune-derived (ADCC and CDC) readouts. To this end, we chose two parental mAbs for each ErbB family member (EGFR, HER2 and HER3) and generated 30 BiXAb™ using the BiXAb™ platform that included all possible combinations and with each Fab positioned on two symmetrical arms at the internal or external position of the BiXAb™.

Recently, the affinity of a single binding interaction between a Fab fragment and its antigen has been defined as the zero-order avidity in the model of response kinetics and thresholds that govern antibody activities (68). Overall in our work, Fabs positioned externally (Fab1 position) and half of those positioned internally (Fab2 position) had an affinity similar to that of the parental mAb (1-3 fold). Thus the BiXAb™ format offers a versatile platform for the rapid generation of tetravalent bispecific antibodies. The tetravalent nature of the BiXAb™ format allowed maintaining the “bivalent avidity” towards the receptors observed for the parental mAbs, which is often not the case with some other BsAb formats composed of monovalent antigen binders (40, 42, 43, 51). On the basis of the recently described dynamic model of antibody/antigen interaction (68), compared to BsAb formats with monovalent antigen binding, the BiXAb™ format, with bivalent antigen-binding sites, should more efficiently induce avidity-mediated interactions, such as antigen-antibody bivalent binding and complex formation (first-order avidity) and clustering of antigen-antibody complexes on the cancer cell surface (second-order avidity). However, we observed a reduction in affinity, referred as zero-order avidity (68), for some of the parental mAbs positioned internally in the BiXAbs™. This could be due to impeded access to the epitope. However, as matuzumab displayed reduced affinity also in the external position in some combinations, this effect may not be linked to the internal positioning in the BiXAb™ format. The importance of Fab positioning in tetravalent BsAbs has been underlined previously (61, 69). Differences in affinity for one of the targets might cause steric hindrance and limit the access to the second target, unless reverse Fab positions are attempted. Our design of an original “long-sized/hinge-derived” linker sequence (i.e. semi-rigid “pseudo-hinge linker” strategy) between Fab1 and Fab2, which was derived from IgG1 and IgA1 hinge sequences that were spliced to create a linker of suitable length, was meant to overcome such structural constraints. It should also be an advantage to obtain bivalent BsAbs showing less flexible, short-sized hinges. Moreover, Wu et al. (69) suggested that variable domains that bind to an antigen of larger molecular size should be preferentially located at the external (N-terminal) position. Here, we did not observe any difference in the molecular mass of the ErbB receptors, thus excluding this factor. Moreover, our data support the hypothesis that the “long-sized/hinge-derived” linker generally allows efficient binding by the internal Fab.

Jarantov et al. demonstrated that binding of the cMETxEGFR bivalent BsAb amivantamab (JNJ-61186372) to tumor cells is guided by the most expressed of its two targeted receptors (70). HER2 and HER3 are moderately expressed in pancreatic cancer, whilst EGFR is overexpressed. Therefore, positioning the EGFR Fab internally in our BiXAb™ would take into account EGFR overexpression to compensate for any potential loss in affinity due to positioning. Moreover, Fabs on the external position might have a greater radius of movement that might favor Fab avidity to the second receptor after binding to the first receptor. This effect should be more pronounced at low receptor density. Our data support this hypothesis because in the best candidates, the anti-HER3 Fab was positioned externally (3Patri) and the anti-EGFR Fab was positioned internally (1Cetux and 1Matu). In addition, their ability to block ligand-induced signaling should be more pronounced against the less expressed target, as shown for amivantamab (70). This might explain why BiXAb™ with 3Patri, 2Trastu and 3Elgem as the external Fab and EGFR as the internal Fab were identified as biologically active in the initial screen.

The selection of Fab arms involving 1Cetu, 1Matu or 3Patri, 3Elgem as the best candidates in our initial screen, irrespective of ligand competition, based on the robust downstream signaling inhibition, and their ability to induce ADCC, suggested that ligand competition was not a prerequisite for BiXAb™ efficacy. Indeed, we previously demonstrated that an allosteric anti-HER3 antibody, non-NRG1 competitive, but with increased binding and biological efficacy in the presence of the ligand, was much more active than an anti-HER3 antibody that competed with the ligand (71).

In pancreatic cancer cells, BiXAb™ targeting ErbB heterodimers inhibited the phosphoproteome and cell proliferation better than homodimer-specific BiXAb™, unlike dual HER2 targeting of non-overlapping epitopes by BsAbs (39) or 2MAbs (72) in breast cancer. This could be explained by the fact that heterodimer-induced signaling is more robust and critical for cell tumorigenicity than homodimer-mediated signaling (73). Moreover, most of the tested BiXAb™ induced stronger inhibition of cell signaling and proliferation than the parental mAbs, but only one third of them displayed greater efficacy than 2MAbs to influence the phosphoproteome profile. In this case, ErbB-specific BiXAb™ could simultaneously inhibit phosphorylation of HER2/HER3, of the underlying kinases, and also of other RTKs, highlighting the strong cross-talk between RTKs (72, 74). Among the BiXAb™ that most strongly inhibited the phosphoproteome in pancreatic cancer cells stimulated with the EGF+NRG1 ligand mix, anti-EGFR/HER3 BiXAb™, including 3Patri-1Cetu-Fc and 3Patri-1Matu-Fc, were consistently more effective. Moreover, EGFR targeting seems to be critical to induce ADCC activation and signaling inhibition, an effect amplified by the external positioning of the anti-HER3 Fab arm.

The BiXAb™ were able to recruit and activate NK cells, in agreement with studies demonstrating that Fabs harboring ErbB valences induce strong ADCC responses *in vitro* and *in vivo* (10, 75, 76). The pivotal role of the anti-EGFR Fab arm to mediate ADCC activation by BiXAb™ and the better capacity of 2MAbs with an EGFR valence to induce ADCC, compared with BiXAb™, could be explained by the higher EGFR expression in the pancreatic cancer cell lines used in the present study. As ADCC requires efficient receptor clustering to be fully operative, the most expressed target is dominant in mediating ADCC. Therefore, an antibody combination would probably be better than a bispecific format because bispecificity requires for cross-linking the binding to a close second receptor (i.e. HER2 or HER3) that is 10-fold to 100-fold less abundant than EGFR. Conversely, when both targets are expressed at similar levels, bispecificity is easier, leading to a clear benefit of BiXAb™ over antibody combinations to favor receptor lattice and subsequent ADCC, as observed for 3Patri-2Trastu-Fc compared with the 3Patri+2Trastu combination in Sw1990 cells.

Our *in vivo* experiments confirmed the interest of co-targeting EGFR and HER3 in pancreatic cancer, in line with previous reports on the anti-EGFR/HER3 BsAb scDb hu225x3-43-Fc in breast and head and neck cancers (52), duligotuzumab (MEHD7945A) (40, 77) in colorectal cancer, and SI-B001 (41) in locally advanced and metastatic tumors. Despite the large molecular weight of the selected BiXAb™ compared to parental MAbs, the 4Fab/IgG1 tetravalent BiXAb™ penetrated deeply within the tumor xenografts and did not remain localized at the tumor periphery or in the stroma. Although pancreatic cancer is a solid tumor with a dense stroma, this observation is in agreement with a previous study showing that the fluorescein-conjugated anti-CEA antibody SGM-101, used to detect residual tumor tissue during surgery, deeply penetrated in pancreatic tumors resected from 12 patients (78).

The combination of ErbB-specific mAbs show synergistic action *in vitro* and *in vivo* in various cancers (10, 18, 31, 79, 80). The therapeutic benefit of BsAbs, compared with 2MAbs, is not limited to the simultaneous targeting of two receptors, resulting in greater anti-tumor effects. They might also preferentially concentrate on tumor cells that express both targets compared with healthy tissues that do not express both receptors or express only one. This may broaden the therapeutic window, as already demonstrated in patients with B lymphoma treated with an anti-CD19/CD22 BsAb (81). Indeed, it has been proposed that BsAb avidity, which is due to their dual binding to two receptors, drives preferential targeting and accumulation of compounds onto tumor cells compared with normal tissues (70). This tumor selectivity depends on BsAb concentration (82, 83), receptor density and internalization/degradation (70, 82, 83), variable domain geometry, and hinge-related flexibility (68, 84). At low BsAb concentration, cell binding is dominated by first- and second-order avidity (68) (bivalent binding of the two receptors). Conversely, the influence of monovalent binding (that depends more on affinity or zero-order avidity (68)) increased as the BsAb concentration was increased (82). As long as the affinity of either arm (monovalent binding) is not too high, a BsAb that targets one receptor that is overexpressed on cancer cells should, through dual-receptor avidity, preferentially bind to tumor cells rather than to healthy cells (70). This could be the case in our pancreatic models, where EGFR density (between 150,000 and 300,000 receptors/cell) is greater than HER2 and HER3 density (5,000-78,000 receptors/cell). Conversely, when density of the two targeted receptors is low or similar (85, 86), the intrinsic affinity of the monovalent BsAb arms predominates over the potential bivalent avidity of 4Fab BsAb to trigger tumor binding selectivity. In addition, the binding (affinity vs avidity) and biological activities of BsAbs are affected by variations in the antibody variable domain geometry, correlated with the hinge region flexibility (84, 87). Our original pseudo-hinge format between Fab1 and Fab2 probably facilitates BiXAb™ structural constraints, and optimizes the distance/orientation of the four paratopes for efficient avidity interactions. Other factors, including IgG subclasses (84), internalization and degradation (51, 58, 84, 88) also can affect the affinity/avidity equilibrium of BsAb interactions, and the subsequent therapeutic index. Moreover, the high affinity of tetravalent BiXAb™ (similar to that of mAbs), their potential ability to bind to FcRn *via* their functional Fc, and their larger size (compared to the parental mAbs) may lead to more favorable pharmacokinetic/pharmacodynamic profiles compared to those of mAbs or combinations. The use of BiXAb™ as a single-drug substance, rather than a combination of mAbs, could be easily translated for industrial implementation and bioprocessing (84).

Although anti-ErbB native antibodies have been used in the clinic for almost 30 years, no BsAb that targets ErbB receptors has been approved yet. All ErbB-specific BsAbs currently in clinical trials are in bivalent format (38, 39, 41, 43), thus reducing their avidity potential for tumor cells. On the basis of the biological activity of these BiXAbs™, after lead optimization, our lead 4Fab/IgG1 tetravalent BiXAb™ 3Patri-1Cetu-Fc, 3Patri-1Matu-Fc, and 3Patri-2Trastu-Fc could improve the therapeutic arsenal against pancreatic cancer.

## Data availability statement

The raw data supporting the conclusions of this article will be made available by the authors, without undue reservation.

## Ethics statement

All *in vivo* experiments were performed in compliance with the French regulations and ethical guidelines for experimental animal studies in an accredited establishment (Agreement No. C34-172-27).

## Author contributions

Conception and design: AP, TC, JC, ER. Development of methodology: ER, VG, CD, GJ, CL, SC-T, EL-C, MC, MJ, PR, LL, GT. Acquisition of data (provided facilities): YB, LG, NP. Analysis and interpretation of data: AP, TC, JC, EAZ, ER. Writing and review of the manuscript: TC, JC, EAZ, ER, LL, GT, NP. Study supervision: AP, P-EG, TC, JC, EAZ. All authors contributed to the article and approved the submitted version.

## Supplementary material

Supplementary Table 1 Raw data of AKT and ERK activation (phosphorylation) in AsPC-1 (file 1), BxPC3 (file 2), CFPAC (file 3), C-PDX P4604 (file 4), Sw1990 (file 5), and SKBR-3 (file 6) cells incubated with the indicated antibodies.

Supplementary Table 2 Raw data of cell viability for BxPC3 (file 1), gemcitabine-resistant BxPC3-GR (file 2), and C-PDX P4604 (file 3) cells incubated with the indicated antibodies.

Supplementary Table 3 Raw data of CD107a degranulation and IFNγ secretion in NK92 cells co-cultured with AsPC1 (file 1), BxPC3 (file 2), CFPAC (file 3), and Sw1990 (file 4) cells incubated with the indicated antibodies.

Supplementary Table 4 Raw data of phosphokinase activation in C-PDX P4604 (file 1), BxPC3 (file 2), and Sw1990 (file 3) cells incubated with the four lead BiXAb™, the corresponding 2MAbs, or 1MAbs.

Supplementary Table 5 Raw data of phosphoreceptor activation in C-PDX P4604 (file 1), BxPC3 (file 2), and Sw1990 (file 3) cells incubated with the four lead BiXAb™, the corresponding 2MAbs or 1MAbs.
