# Supplementary figures and images for: Design and selection of optimal ErbB-targeting bispecific antibodies in pancreatic cancer
Source: Front Immunol. 2023 Apr 20;14:1168444. doi: 10.3389/fimmu.2023.1168444 (PMC10157173; doi:10.3389/fimmu.2023.1168444)

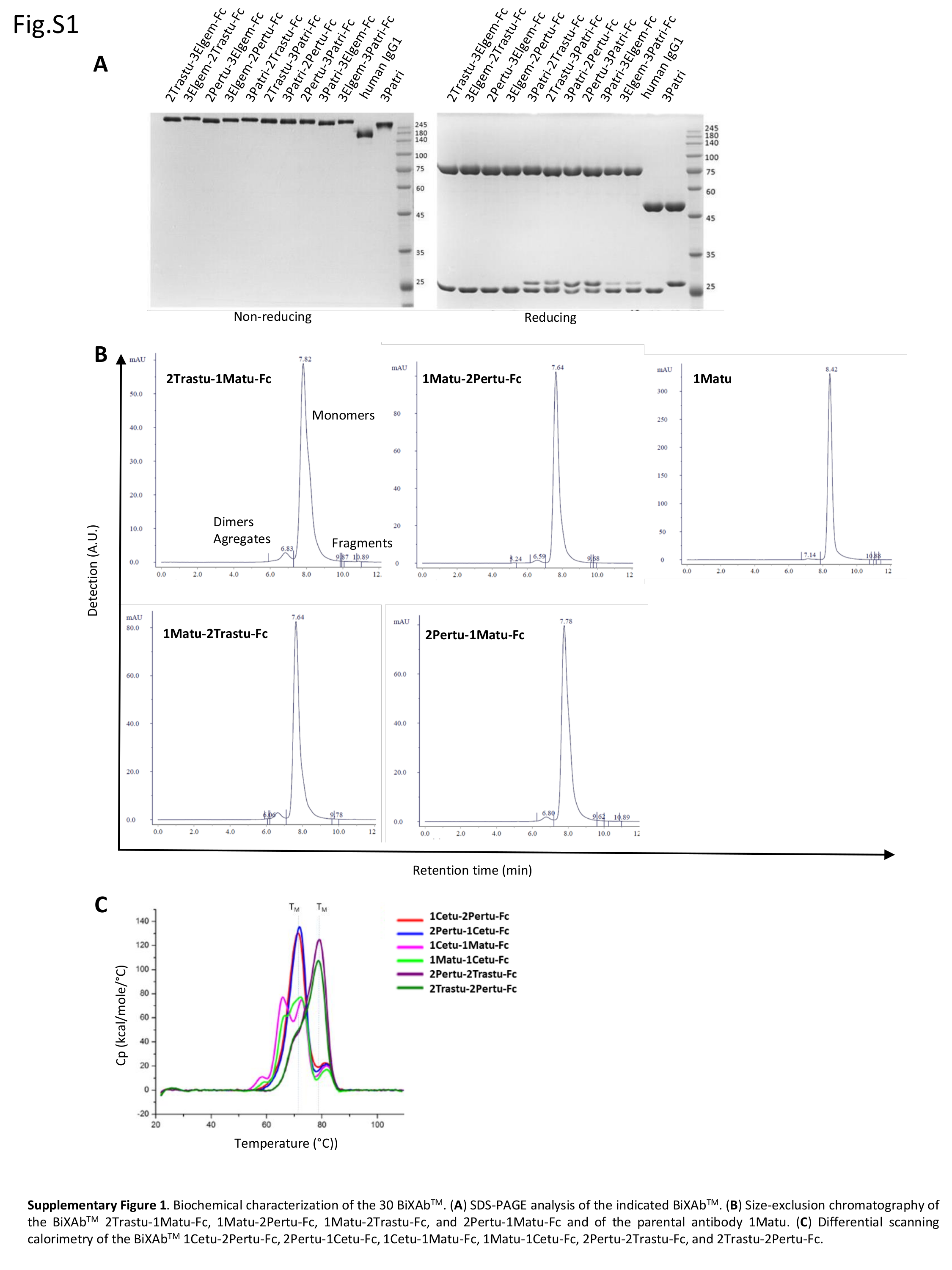

Supplement: Supplementary file 6 [file Image_1.tif]

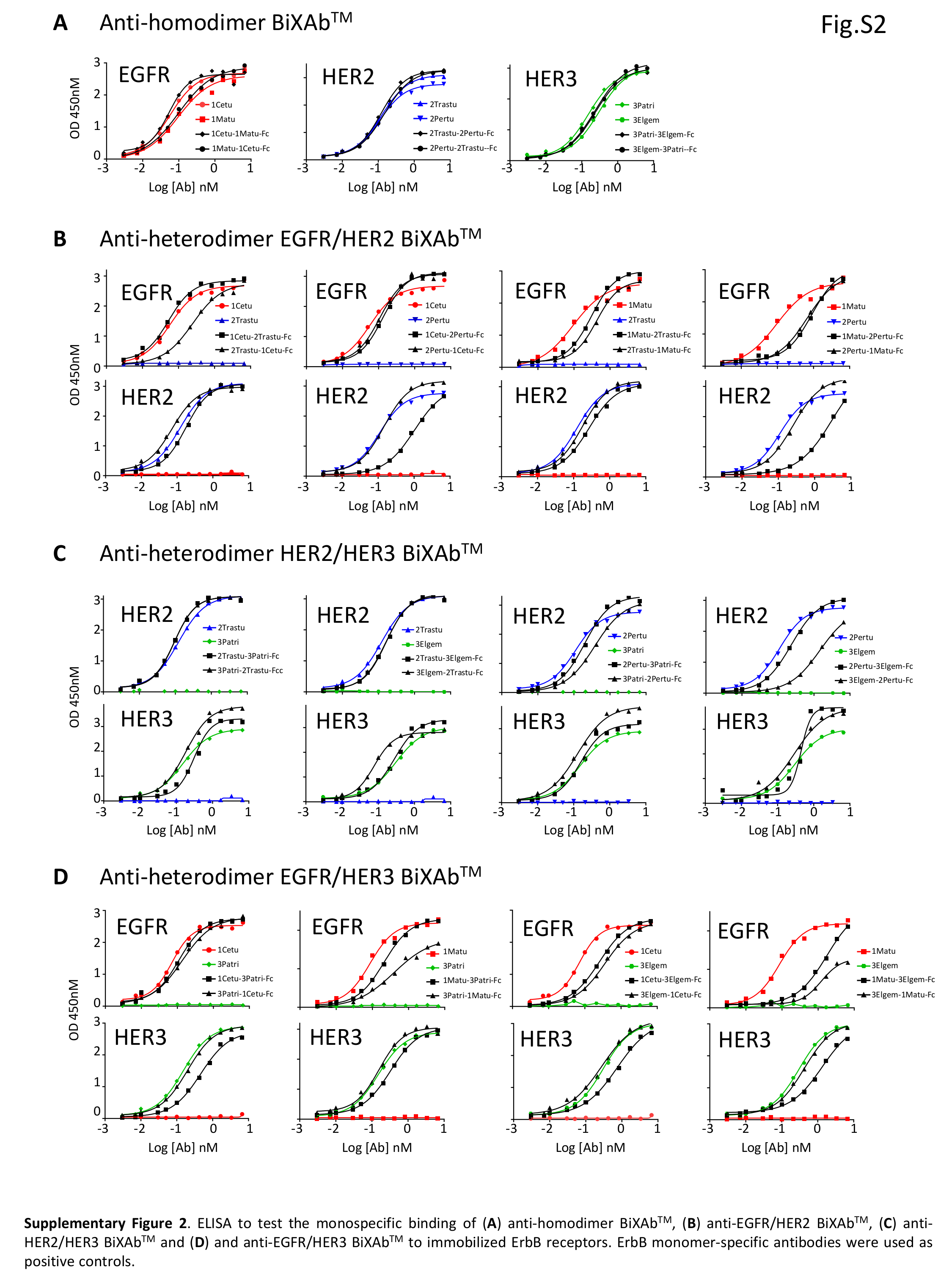

Supplement: Supplementary file 7 [file Image_2.tif]

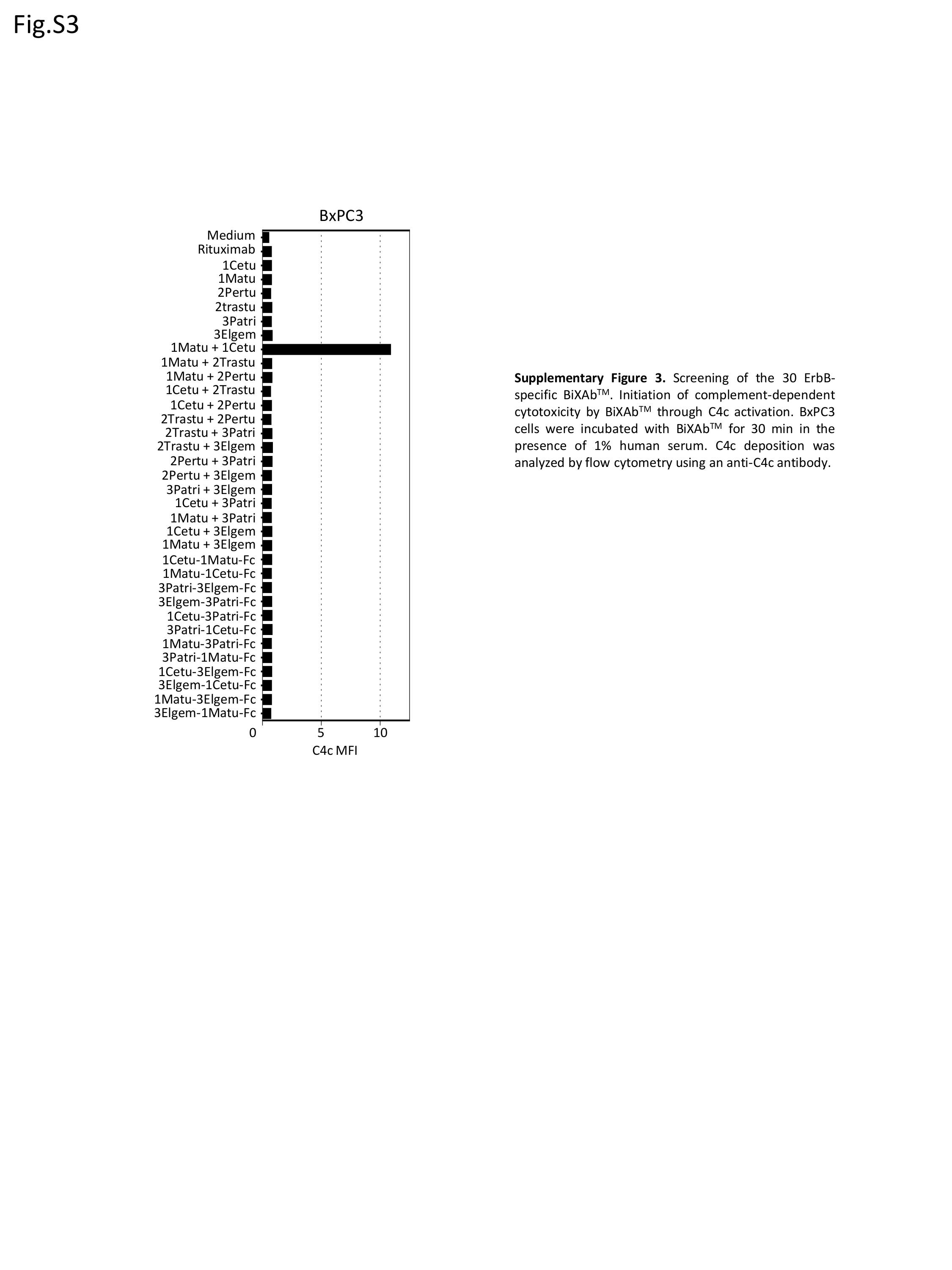

Supplement: Supplementary file 8 [file Image_3.tif]

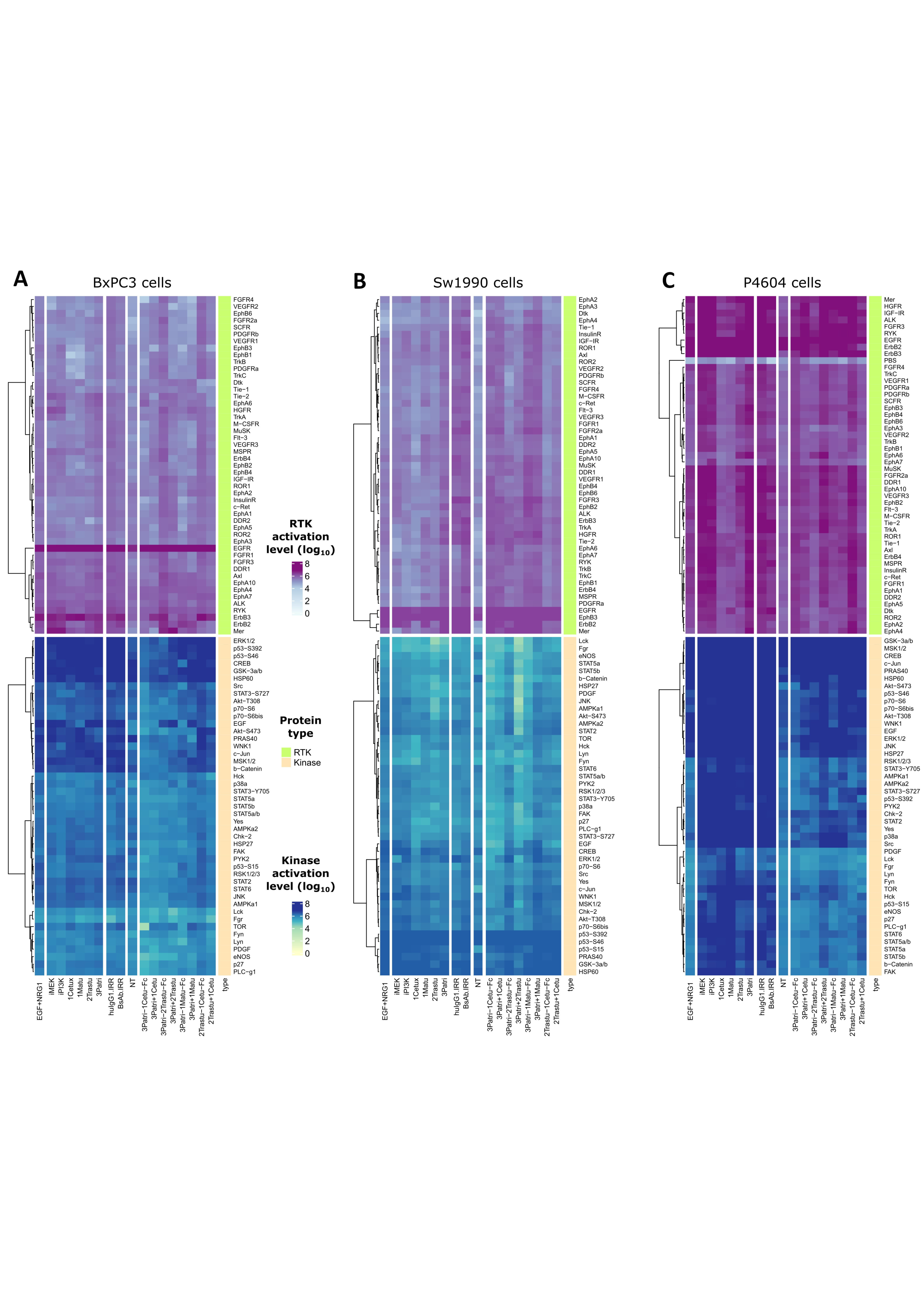

Supplement: Supplementary file 9 [file Image_4.tif]

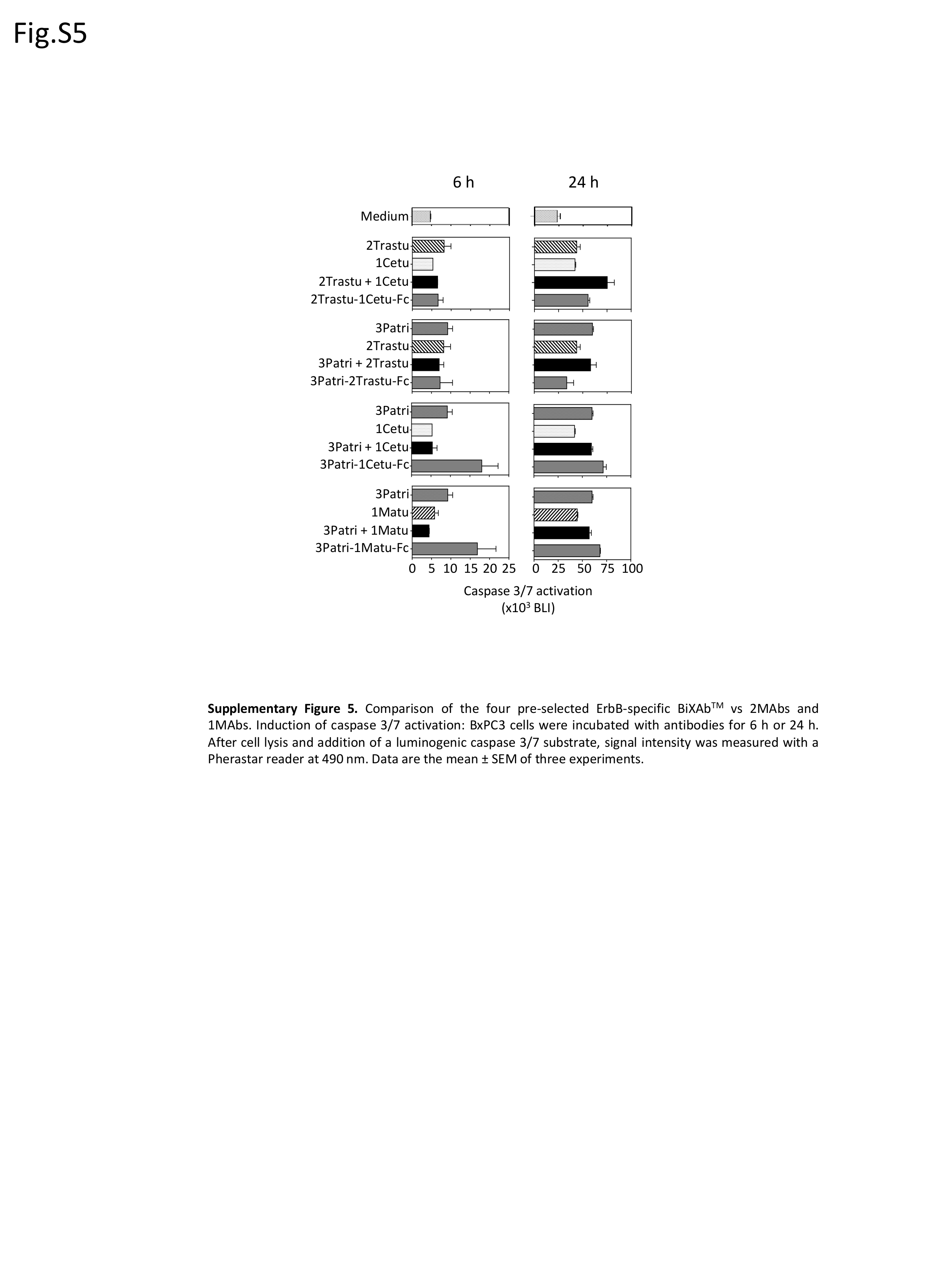

Supplement: Supplementary file 10 [file Image_5.tif]

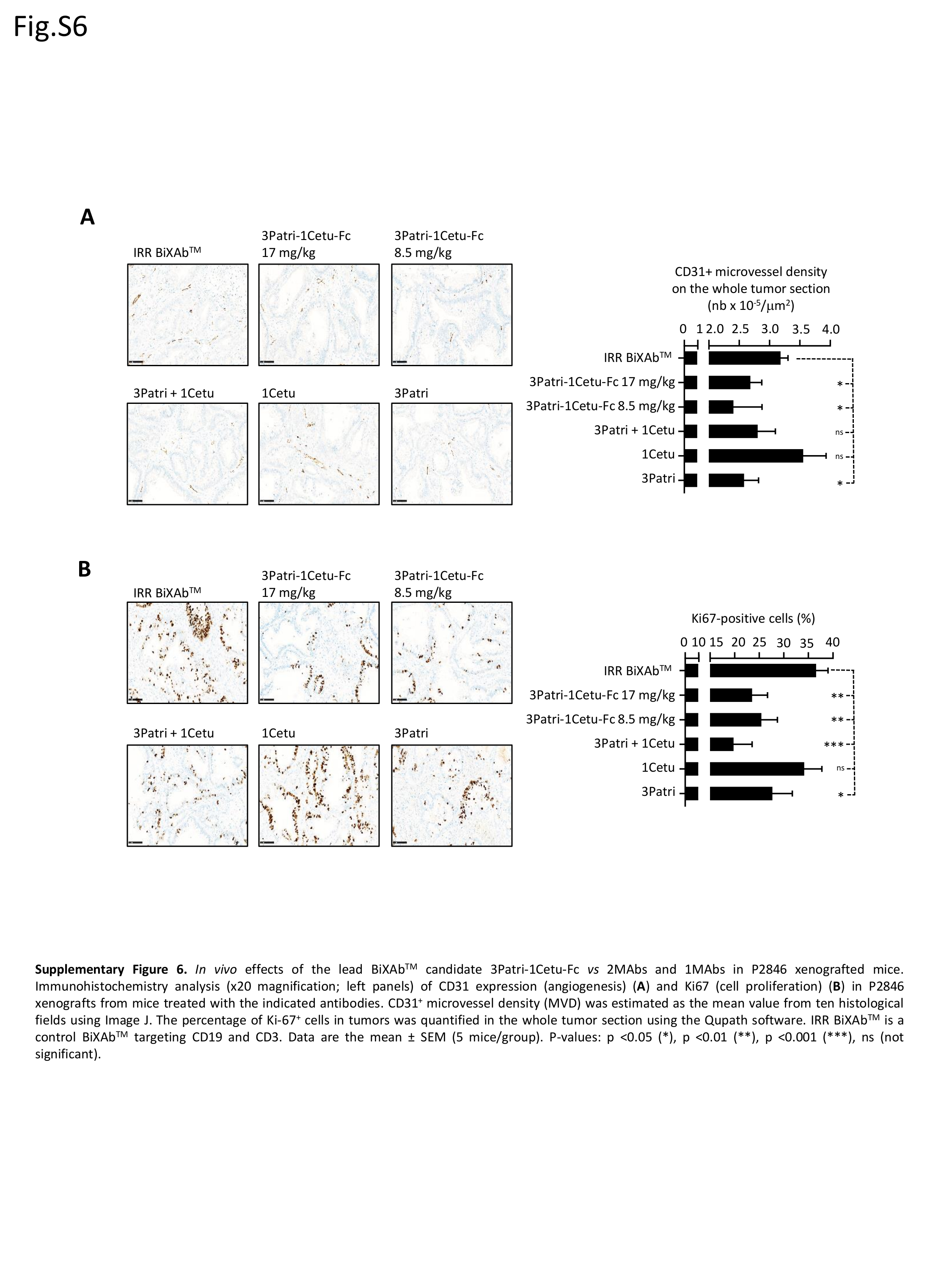

Supplement: Supplementary file 11 [file Image_6.tif]

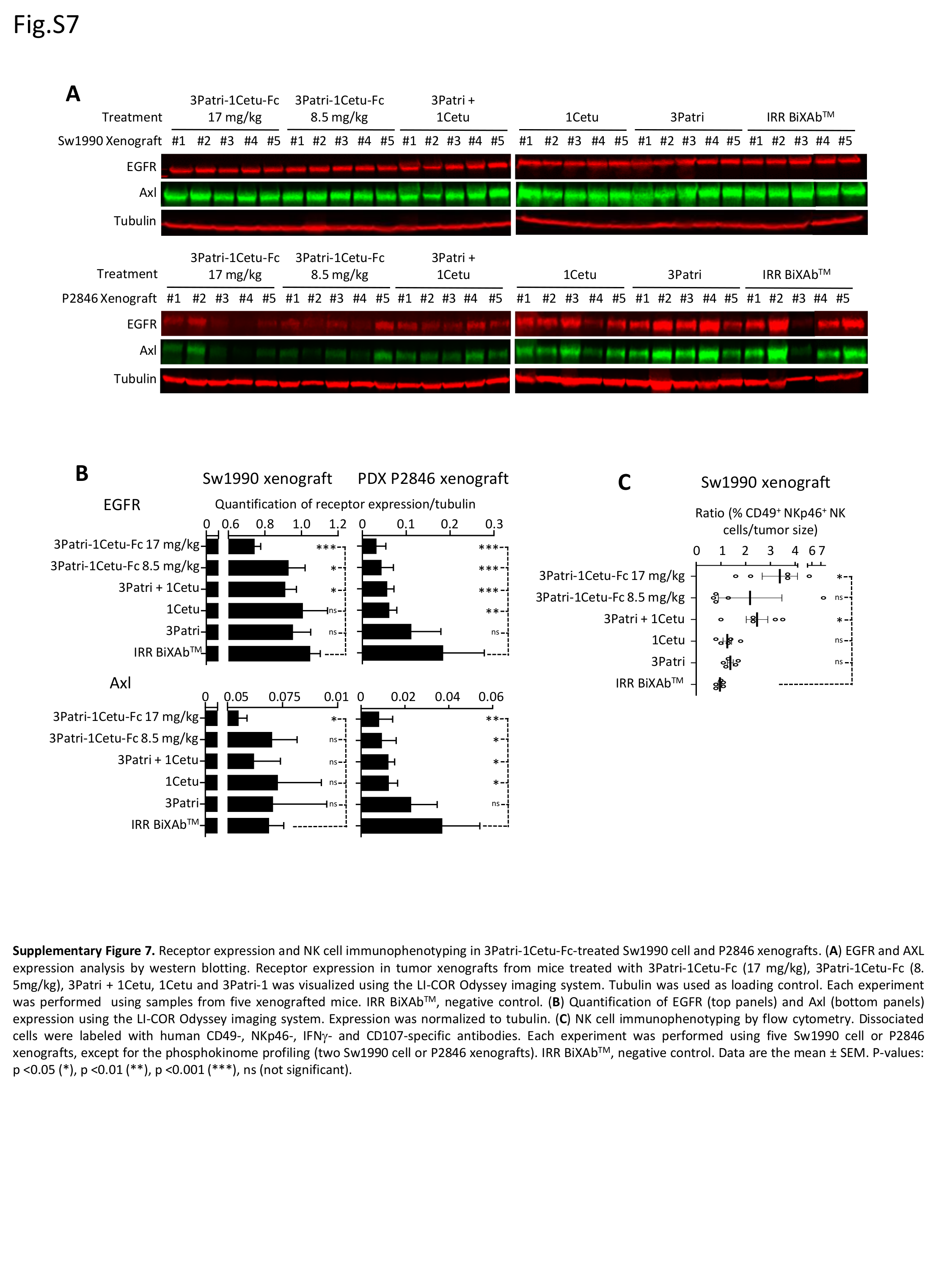

Supplement: Supplementary file 12 [file Image_7.tif]
